# The genetic spectrum of a cohort of patients clinically diagnosed as Parkinson’s disease in mainland China

**DOI:** 10.1038/s41531-023-00518-9

**Published:** 2023-05-17

**Authors:** Yi-Min Sun, Xin-Yue Zhou, Xiao-Niu Liang, Jin-Ran Lin, Yi-Dan Xu, Chen Chen, Si-Di Wei, Qi-Si Chen, Feng-Tao Liu, Jue Zhao, Yi-Lin Tang, Bo Shen, Lin-Hua Gan, Boxun Lu, Zheng-Tong Ding, Yu An, Jian-Jun Wu, Jian Wang

**Affiliations:** 1 Department of Neurology and National Research Center for Aging and Medicine & National Center for Neurological Disorders, State Key Laboratory of Medical Neurobiology, Huashan Hospital, Fudan University, Shanghai, China; 2 Human Phenome Institute, Zhangjiang Fudan International Innovation Center, MOE Key Laboratory of Contemporary Anthropology, Fudan University, Shanghai, China; 3 Neurology Department at Huashan Hospital, State Key Laboratory of Medical Neurobiology and MOE Frontiers Center for Brain Science, Institutes of Brain Science, School of Life Sciences, Fudan University, Shanghai, China

**Keywords:** Clinical genetics, Parkinson's disease

## Abstract

So far, over 20 causative genes of monogenic Parkinson’s disease (PD) have been identified. Some causative genes of non-parkinsonian entities may also manifest with parkinsonism mimicking PD. This study aimed to investigate the genetic characteristics of clinically diagnosed PD with early onset age or family history. A total of 832 patients initially diagnosed with PD were enrolled, of which, 636 were classified into the early-onset group and 196 were classified into the familial late-onset group. The genetic testing included the multiplex ligation-dependent probe amplification and next generation sequencing (target sequencing or whole-exome sequencing). The dynamic variants of spinocerebellar ataxia were tested in probands with family history. In the early-onset group, 30.03% of patients (191/636) harbored pathogenic/likely pathogenic (P/LP) variants in known PD-related genes (*CHCHD2*, *DJ-1*, *GBA (heterozygous)*, *LRRK2*, *PINK1*, *PRKN*, *PLA2G6*, *SNCA* and *VPS35*). Variants in *PRKN* were the most prevalent, accounting for 15.72% of the early-onset patients, followed by *GBA* (10.22%), and *PLA2G6* (1.89%). And 2.52% (16/636) had P/LP variants in causative genes of other diseases (*ATXN3, ATXN2, GCH1, TH, MAPT, GBA (homozygous)*). In the familial late-onset group, 8.67% of patients (17/196) carried P/LP variants in known PD-related genes (*GBA (heterozygous), HTRA2, SNCA*) and 2.04% (4/196) had P/LP variants in other genes (*ATXN2, PSEN1, DCTN1*). Heterozygous *GBA* variants (7.14%) were the most common genetic cause found in familial late-onset patients. Genetic testing is of vital importance in differential diagnosis especially in early-onset and familial PD. Our findings may also provide some clues to the nomenclature of genetic movement disorders.

## Introduction

Parkinson’s disease (PD) is a progressive neurodegenerative movement disorder with bradykinesia, rigidity and rest tremor as the cardinal motor symptoms. Genetic research on PD has achieved significant advance in recent years^1^. So far, pathogenic/likely pathogenic (P/LP) variants in over 20 genes that lead to Mendelian inheritance have been identified in PD with various evidence levels^2–4^ (defined as known PD-related genes in the study), accounting for 3%–5% of all PD patients^1^ and 7%–9% of PD patients with early onset age (<50 years old) or family history^5–7^.

Based on the phenotypic heterogeneity, patients with P/LP variants in causative genes of other diseases, such as *ATXN2* in spinocerebellar ataxia (SCA)^8^, *GCH1* in dopamine responsive dystonia (DRD)^9^ or *MAPT* in frontotemporal lobar degeneration (FTLD)^10^, might present with PD-like symptoms in the early stage or even in the late disease course^11^ and might be clinically misdiagnosed as PD.

Large scale genetic studies of PD have been carried out in different ethnic populations in recent years^5–7,12–16^. The frequency of P/LP variants ranged from 1.4 to 14%, with higher rate of 4.1–33.9% in early-onset Parkinson’s disease (EOPD) patients or patients with family history in different studies. Though P/LP variants in *PRKN*, *LRRK2* and *GBA* were commonly detected, the genetic architecture differs in different ethnicities. Two large genetic studies of Chinese PD patients have been reported recently^5,15^. One study included familial PD, EOPD and sporadic PD and focused on 23 known PD-associated genes while the other included EOPD and focused on 26 PD-related genes and 20 other genes linked to neurodegenerative and lysosome diseases.

Here we conduct another large-scale genetic testing in Chinese Han PD patients. We investigated the 116 disease causative genes (including 22 known PD-related genes) in a cohort of clinically diagnosed PD patients with early age at onset (AAO) or family history to identify the genetic spectrum and the nomenclature of genetic movement disorders besides the genetic screen strategy.

## Results

### The demographic and the clinical features of the patients initially diagnosed as PD

The median AAO of the early-onset group and familial late-onset group was 39.00 (12.00) and 59.00 (10.00) years, respectively [Median (interquartile range (IQR)), *p* < 0.0001], and the disease duration was 46.00 (67.00) and 45.00 (57.00) months (*p* = 0.1371), respectively (Supplementary Table 1). The patients with family history in early-onset group account for 28.62% (181/636). There is significant difference between the two groups regarding sex (*p* = 0.0306) and education years (*p* = 0.0001). All the participants were self-reported Chinese Han ethnic.

When divided by family history, 455 patients (54.65%) were sporadic early-onset and 377 (45.31%) had family history (269 with autosomal dominant inheritance and 108 with autosomal recessive inheritance). The median AAO of sporadic early-onset patients and familial patients was 39.00 (12.00) and 50.00 (19.00) years, respectively (*p* = 0.0001), and the median disease duration was 44.00 (60.00) and 48.00 (71.00) months, respectively (*p* = 0.5297).

### Distribution of causative genes in patients initially diagnosed as PD

P/LP variants responsible for the parkinsonian phenotype were found in 17 genes, including 10 known PD-related genes (*CHCHD2*, *DJ-1*, *GBA*, *HTRA2*, *LRRK2*, *PINK1*, *PRKN*, *PLA2G6*, *SNCA* and *VPS35)* and 7 causative genes of other diseases (*ATXN2*, *ATXN3*, *DCTN1*, *GCH1*, *MAPT*, *PSEN1 and TH*). P/LP variants in each patient and their genomic locations were reported in Supplementary Table 2 and Supplementary Fig. 1.

**Fig. 1:**
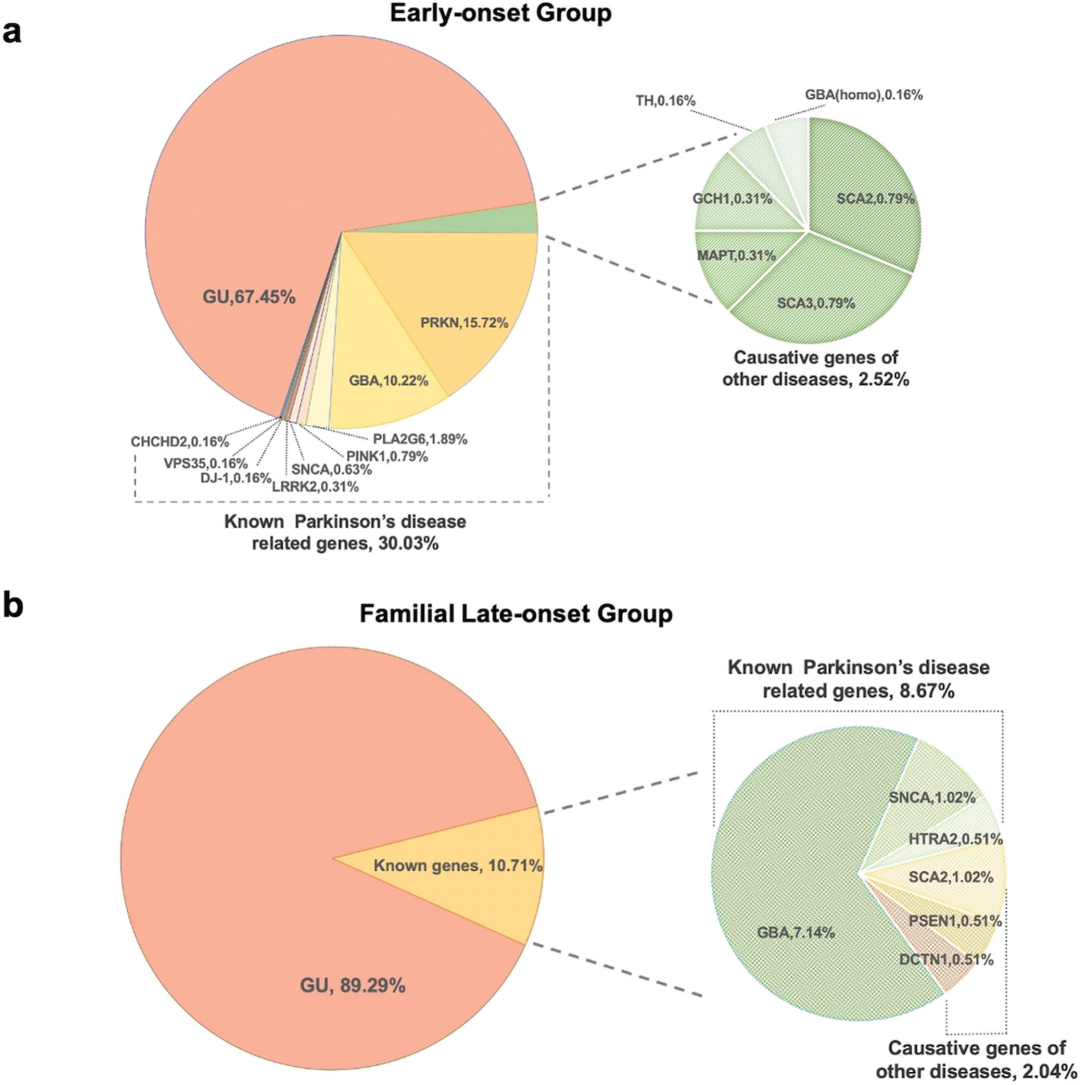
The frequency of causative genes in clinical diagnosed Parkinson’s disease. The frequency of causative genes in the early-onset patients (**a**) and familial late-onset patients (**b**) with clinical diagnosis of Parkinson’s disease.

P/LP variants were found in 32.55% patients (207/636) in early-onset group and 10.71% (21/196) in familial late-onset group (Fig. 1). In early-onset group, 191 probands (30.03%) carried P/LP variants in known PD-related genes. *PRKN* was the most prevalent one, with 100 patients bearing two variants (15.72%, 33 patients without family segregation analysis), followed by *GBA* heterozygous variants (*n* = 65, 10.22%), *PLA2G6* (*n* = 12, 1.89%), *PINK1* (*n* = 5, 0.79%, 1 patient without family segregation analysis), *SNCA* (*n* = 4, 0.63%) (Supplementary Fig. 2), *LRRK2* (*n* = 2, 0.32%), *DJ-1* (*n* = 1, 0.16%), *VPS35* (*n* = 1, 0.16%), and *CHCHD2* (*n* = 1, 0.16%). Sixteen probands (2.52%) carried P/LP variants in causative genes of other diseases. Among them, 10 patients carried dynamic variants of SCA (five with *ATXN3*, five with *ATXN2*), as well as three patients with variants in DRD-related genes (two with *GCH1*, one with *TH*), two patients bearing *MAPT* p.Asn279Lys related with FTLD, and one carrying homozygous *GBA* p.Phe76Val variant causing Gaucher’s disease, respectively.

In familial late-onset group, 17 patients (17/196, 8.67%) carried P/LP variants in known PD-related genes, lower than the early-onset group. Among them, 14 probands carried *GBA* heterozygous variants (7.14%), two probands carried *SNCA* duplication (1.02%) and one carried *HTRA2* c.940-1G > C variant (0.51%). Four probands (2.04%) bore P/LP variants in causative genes of other diseases, including two with spinocerebellar ataxia type 2 (SCA2), one with Alzheimer’s disease (AD) (*PSEN1* p.Leu282Arg), and one with Perry syndrome (*DCTN1* p.Tyr78His).

Twenty-nine novel P/LP single nucleotide variants and short insertions/deletions were found in the cohort, with 13 in *PRKN* (NM_004562.3, p.Gln63Ter, p.Pro73Argfs*8, p.Val117Glyfs*9, p.Thr217Profs*8, p.Glu309Ter, p.Cys337Tyr, p.Tyr372Valfs*2, p.Ala406Glyfs*168, p.Glu426Gly, p.Trp462Ter, c.535-2A > C, c.535-3A > G, c.619-2A > G), eight in *GBA* (NM_000157.4, p.Ile158Serfs*42, p.Met162Val, p.Leu199Aspfs*62, p.Gly228Arg, p.Pro240Leu, p.Val414Leu, p.Trp417Ter, p.Asp419Asn), four in *PLA2G6* (NM_003560.4, p.Thr518Ala, p.Phe549Ile, p.Ala633Val, p.Ala640Thr), two in *PINK1* (NM_032409.3, p.Val59Serfs*48 and p.Asn521Tfs*40), one in *HTRA2* (NM_001321727.1, c.940-1G > C), and one in *DJ-1* (NM_007262.5, p.Val71del) (Supplementary Table 2 and Supplementary Fig. 1). In addition, one novel triplication of exon 2–4 of *PRKN* was also found.

The mutational frequency of all the screened genes and the genotypes were described in Supplementary Materials.

### The clinical characteristics related to the known PD-related genes in the cohort

Comparing with genetic undefined (GU) patients in early-onset group, *PRKN* patients had an earlier AAO, longer disease duration and less levodopa equivalent daily dosage (LEDD), and tended to have more preserved olfactory function and cognitive function in domains of executive function, language, and attention. After adjusting for confounders (age, sex, education, disease duration, and LEDD), all the significance remained. More females and shorter disease duration at baseline were found in patients bearing heterozygous *GBA* variants. More significant olfactory dysfunction was observed in *GBA p*atients, both before and after adjusting for confounders. Patients with P/LP variants in *PLA2G6* had an earlier AAO, younger age at examination. They showed more severe motor dysfunction with higher Unified Parkinson’s Disease Rating Scale (UPDRS) III score. They were more affected in non-motor symptoms with higher Non-Motor Symptoms Questionnaire (NMSQ) scores and more depression. They also had significantly more decreased quality of life and worse cognitive function in all the 5 subdomains than GU patients both before and after adjustment (Table 1, Supplementary Table 3). P/LP *PINK1* variants were detected in 5 patients. The median age at examination of *PINK1* variant carriers was 31.00 (21.50) years. From the clinical observation, patients with *PINK1* variants usually had a young onset age (30.00 (21.00)), and preserved olfactory function evaluated by Sniffin’ Sticks Screening 12 test (SSST-12) (9.00 (4.25)). Six patients carried *SNCA* variants. The median age at examination of *SNCA* variant carriers was 51.50 (23.50) years. The wide range of AAO (22 to 62 years) and worse scores in SSST-12 (2.00 (4.00)) were observed in *SNCA* patients (Supplementary Table 4).

**Table 1:** Clinical features of the GU-EOPD patients, and the EOPD patients with pathogenic/likely pathogenic variants in *PRKN*, *GBA*, and *PLA2G6*.

Clinical features	GU-EOPD (*N* = 429)	*PRKN* (*N* = 100)	*GBA* (*N* = 65)	*PLA2G6* (*N* = 12)	*P*		
					*PRKN*	*GBA*	*PLA2G6*
Sex, female (%)	160 (37.30)	41 (41.00)	36 (55.38)	5 (41.67)	0.4920	0.0055**	0.7576
Education, years	12.00 (6.00)	12.00 (6.00)	11.00 (7.00)	12.50 (8.00)	0.9253	0.6765	0.6058
AAO, years	41.00 (9.00)	27.00 (11.00)	42.00 (8.00)	27.00 (8.00)	0.0001**	0.4419	0.0001**
Age at examination, years	45.00 (11.00)	37.00 (13.50)	45.00 (10.00)	30.00 (4.50)	0.0001**	0.7449	0.0001**
Disease duration, month	43.00 (60.00)	93.00 (123.50)	31.00 (54.00)	33.00 (39.00)	0.0001**	0.0314*	0.3928
LEDD, mg	450.00 (400.00)	350.00 (400.00)	475.00 (375.00)	350.00 (400.00)	0.0034*	0.1294	0.8828
UPDRS III, score (Med off)	27.25 (21.00)	30.00 (17.00)	28.50 (20.00)	39.00 (15.75)	0.2598	0.9394	0.0148*^a^
**Non-motor features**							
BDI, score	12.00 (12.00)	14.00 (13.00)	11.00 (17.00)	23.00 (12.00)	0.1838	0.5385	0.0001**^a^
PDQ39, score	29.00 (34.00)	38.00 (32.00)	28.00 (46.00)	59.00 (44.00)	0.0542	0.5028	0.0012**^a^
NMSQ, score	10.00 (10.00)	9.00 (5.00)	9.00 (10.00)	15.00 (6.50)	0.3362	0.3336	0.0266*
ESS, score	6.00 (6.00)	5.00 (5.00)	5.00 (5.00)	7.00 (4.00)	0.7024	0.4791	0.3527
RBDSQ, score	3.00 (3.00)	3.00 (3.00)	3.00 (3.00)	3.00 (0.00)	0.4316	0.9808	0.7485
SSST-12, score	6.00 (3.00)	8.50 (3.00)	5.00 (3.00)	5.00 (4.00)	0.0001**^a^	0.0003**^a^	0.1838^a^
Cognitive domains							
Memory	−0.07 (1.08)	0.07 (1.08)	−0.08 (1.18)	−1.10 (0.86)	0.2463	0.7969	0.0017**^a^
Visuospatial function	0.22 (0.76)	0.21 (0.82)	0.06 (1.22)	−0.78 (1.79)	0.8576	0.2634	0.0022**^a^
Language	−0.02 (1.06)	0.27 (1.29)	−0.25 (1.13)	−1.22 (2.54)	0.0034**^a^	0.2124	0.0434*^a^
Attention	0.09 (0.98)	0.45 (0.93)	0.30 (1.23)	−0.47 (1.76)	0.0001**^a^	0.1113	0.0308*^a^
Executive function	0.08 (0.78)	0.31 (0.83)	0.13 (0.82)	−0.36 (1.69)	0.0001**^a^	0.4364	0.0515^a^
MMSE, score	28.00 (2.00)	29.00 (1.00)	28.50 (3.00)	28.00 (2.00)	0.3661	0.9885	0.0722^a^

The continuous data are presented as median (interquartile range (IQR)), and the categorical data are presented as *n* (%).

*AAO* age at onset, *EOPD* early-onset Parkinson’s disease, *GU* genetically undefined, *BDI* beck depression inventory, *ESS* Epworth Sleepiness Score, *LEDD* Levodopa equivalent dose daily, *MMSE* mini mental state examination, *NMSQ* non-motor symptoms questionnaire, *PDQ39* 39-item Parkinson’s disease questionnaire, *RBDSQ* rapid-eye-movement sleep behavior disorder screening questionnaire, *SSST-12* Sniffin’ Sticks screening 12 test, *UPDRS* Unified Parkinson’s Disease Rating Scale.

^a^Significant after adjustment for age, gender, education, disease duration, and levodopa dose equivalents.

*P*: Comparison between the early-onset group with variants within a specific gene and GU-EOPD group. *P < 0.05, **P < 0.001.

### The clinical characteristics related to the causative genes of other diseases in the cohort

As for the patients carrying P/LP variants in causative genes of other diseases, all the patients met the clinical diagnostic criteria of PD at the first clinical visit. All the affected family members of the probands with family history were diagnosed or suspected with PD. No cerebellar signs were observed in all of the SCA patients at the baseline visit, and only one SCA2 patient showed mild nystagmus at follow-up. As for the noteworthy features, three out of seven SCA2 patients had dystonia, with one patient having foot dystonia and blepharospasm at the first year of disease and 2 patients developing blepharospasm, and spastic torticollis/upper limb dystonia respectively during follow-up. One spinocerebellar ataxia type 3 (SCA3) patient had peripheral neuropathy and one patient with *PSEN1* variant had a medical history of epilepsy and stroke. Among the 12 patients followed up in our center, 10 patients still met the clinical diagnostic criteria of PD with the disease duration from 4 to 15 years, and the other two with *MAPT* variant developed progressive supranuclear palsy (PSP) or frontotemporal dementia like symptoms at the third and fourth year of disease (Supplementary Table 5).

## Discussion

**Fig. 2:**
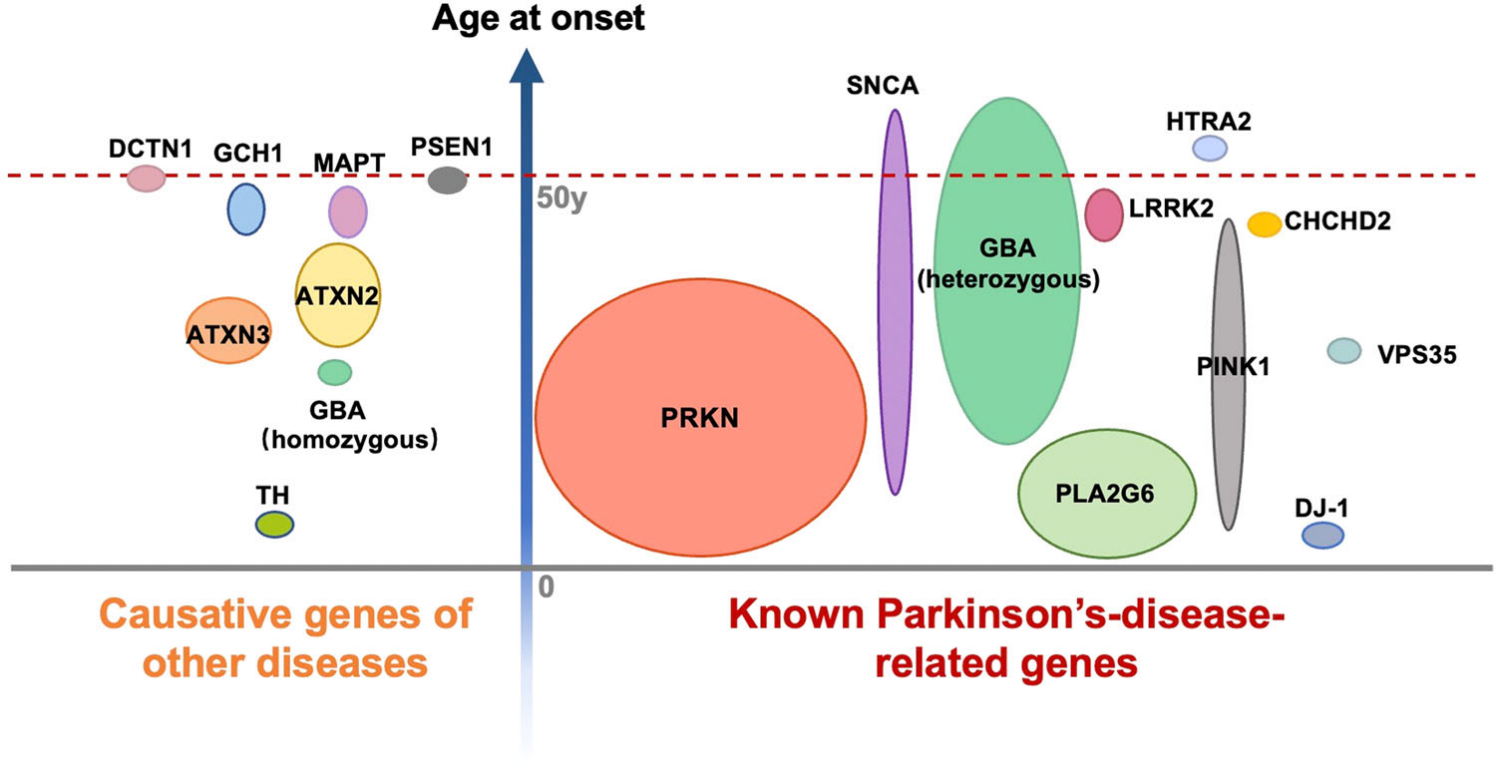
The genetic architecture of clinical diagnosed Parkinson’s disease in the study. The vertical axis represents the age at onset. The size of a certain gene illustrated in the figure represents the frequency identified in our study.

In the current study, the genetic spectrum was investigated in a cohort of clinically diagnosed PD patients from mainland China. We found 26.92% patients (224/832) carrying P/LP variants in known causative genes in a cohort of patients with an early AAO or family history. Of note, twenty patients (20/832, 2.15%) carried P/LP variants in causative genes of other diseases, including *ATXN2*, *ATXN3*, *GCH1*, *MAPT*, *PSEN1*, *TH*, *GBA* (homozygous variants) and *DCTN1*. The genetic architecture in our cohort and its relationship with clinical diagnosis and AAO were summarized in Fig. 2^17,18^.

For the known PD-related genes, we found a higher frequency of P/LP variants in early-onset patients (30.03%) than familial late-onset patients (8.67%), this finding is consistent with previous reports that the genetic factors play a major role in the disease onset in EOPD^5^. The frequency of P/LP variants (8.67%) was much lower in familial late-onset patients comparing with that in all the included familial patients regardless of onset age (27.05%), though all the probands had family history of parkinsonism. Notably, two third late-onset patients in the cohort had a family history of late-onset PD. The familial aggregation of late-onset PD might partly be explained by the genetic burden of rare variants or common variants, the similar environmental exposures in one family, and the prevalence with ageing, rather than a single strong genetic effector^19^.

The frequency of causative genes or the P/LP variant spectrum within a certain gene varied among different geographical regions and populations^12,20,21^. *LRRK2* variants were reported as the leading genetic cause of PD in European population, with *LRRK2* G2019S explaining about 5–6% familial PD and 1% sporadic cases^13^. The frequency of *LRRK2* G2019S in PD can surge to almost 14% in Ashkenazi Jews population^5^. However, no *LRRK2* G2019S were identified in our cohort in line with previous reports in Asian population^22^. In Chinese EOPD and familial PD patients, P/LP variants in *PRKN* were the most prevalent with 4.3–5.7% in different studies, followed by heterogenous *GBA* variants (2.1–7.2%)^5–7,15^. The P/LP variants of *PINK1* (0.4–1.2%), *PLA2G6* (0.3–0.6%), *SNCA* (0.2–0.7%), *LRRK2* (0.4–2.0%), *VPS35* (0.06–0.3%) were reported with a lower frequency. However, in this study, the proportion of patients carrying *PRKN* (15.72%), *GBA* (9.50%) and *PLA2G6* (1.89%) variants was higher than previously reported, while the proportion of patients carrying the other PD causative genes was similar. Accordingly, the frequency of known PD-related genes excluding *GBA* was 19.81% in early-onset group, higher than other findings (11.6%^15^, 9.3%^6^, 8.74%^5^, 7.5%^7^). It could be interpreted to the distribution of AAO and the selective bias of a single center of tertiary hospital. The percentage of patients with AAO ≤ 30, 30 < AAO ≤ 40, and 40 < AAO ≤ 50 was 19.6%, 37.2%, 45.1% in our cohort, with much more patients with younger AAO than those in Zhao’s study (6.6%, 19.6% and 73.8%)^5^. This may explain the higher molecular diagnosis rate of our study, since the lower AAO was related with a higher molecular diagnosis rate^5^.

Dynamic variants of *ATXN2* and *ATXN3* explained 1.4% of familial PD in this study, which indicated the importance of genetic testing of dynamic variants in patients with parkinsonian symptoms. Though autosomal dominant cerebellar ataxia was recognized as the predominant phenotype of SCA2, parkinsonism was also common in SCA2^23^. The SCA2 patients predominantly manifesting parkinsonism might be reminiscent of PD patients and should be detected by genetic testing^24,25^. Similarly, parkinsonian phenotype was also frequently described in SCA3, albeit less prevalent than ataxia^26,27^. Intrafamilial phenotypic heterogeneity was found in reported SCA3 cases with parkinsonism^28^, but the affected family members of four SCA3 probands in this study were all PD phenotype, resulting in further setbacks to the exact diagnosis.

SCA2 or SCA3 patients with parkinsonism being the dominant symptoms could have unremarkable findings in brain magnetic resonance images (MRI)^25,29^, which indicated the brain MRI may or may not be the clues to differential PD from SCA. One patient with SCA3 dynamic variant had peripheral neuropathy, which was commonly observed in ataxia predominant SCA2 and SCA3^30,31^ and was also reported in parkinsonism dominant SCA2 and SCA3^26,32^. Though the percentage of SCA dynamic variants in patients with parkinsonism plus peripheral neuropathy was unknown, we suggested besides family history, peripheral neuropathy can be another important hint to the SCA diagnosis.

The P/LP variants of *GCH1* were initially detected in the patients with DRD. Besides classic phenotype of DRD, the broadened clinical spectrum of *GCH1* can include adult-onset parkinsonism, focal dystonia, DRD-simulating cerebral palsy or spastic paraplegia^33^. The P/LP variants of *GCH1* were later reported to be associated with DRD and PD in different family members in the same pedigree, and even with sporadic PD patients^9,34,35^, indicating the *GCH1-* related mechanism might contribute to the pathogenesis of PD and tend to increase the risk of PD^34^. The patients in our study showed PD-like symptoms. Significant sleep benefit and good response to levodopa treatment was observed but no dystonia. They might be PD or DRD since the two *GCH1* variants were reported both in DRD and PD^2,34,35^. The positron emission computed tomography (PET) for dopamine transporter could be helpful to the diagnosis, which, however, was not applied in the patients.

Biallelic *TH* and *GBA* variants were identified as the genetic cause of DRD and Gaucher’s disease. For phenotypic spectrum, patients with homozygous/compound heterozygous variants of *TH* or *GBA* could also present PD-like characteristics^36–38^. *DCTN1* was initially identified as the genetic cause of Perry syndrome, but adult-onset (atypical) parkinsonism was later found prominent in patients bearing *DCTN1* variants^39^. Patients with *MAPT* or *PSEN1* variants usually present with FTLD and AD respectively, but can have prominent parkinsonism^10,40^. In the early disease stage, these patients might manifest characterized symptoms of PD and be misdiagnosed as PD^10,40–43^. With the progression of the diseases, more atypical symptoms emerge which indicate other diagnosis instead of PD. Parkinsonism is one of the core phenotypes of the frontotemporal dementia and parkinsonism linked to chromosome 17 (FTDP-17) spectrum caused by *MAPT* variants^10^, which should be differentiated from PD in the first place especially in cases with the autosomal dominant inherited family history. Besides, the atrophy pattern on brain MRI may give clues to the diagnosis of FTLD or AD, but the genetic testing should be implemented early whenever possible.

Our work may provide important implication for the application and interpretation of genetic testing in clinically diagnosed PD patients, especially in Chinese Han population. The genetic testing will improve the diagnostic accuracy of PD in the early-onset and familial patients, especially those with onset age below 50. Besides known PD-related genes, causative genes of other diseases should also be attached importance during interpretating the results of genetic testing, as they were also the indispensable genetic contributor to the PD-like phenotype.

Our findings would also refine the patient recruitment for clinical trials. The “one-size-fits all” patient recruitment approach contributed a lot to the high rate of failure in previous PD clinical trials^44,45^. The genetic status determined by proper genetic testing can lead to quick access to eligible candidates for new treatment options. Genetically targeted clinical trials and the implementation of personalized medicine may bring new opportunities to PD disease-modifying therapy.

Due to the clinical heterogeneity of the neurogenetic diseases, the broad and complex phenotypes-including the parkinsonian characteristics, can be associated with a specific causative gene, sometimes it is a causative gene of other disease. Meanwhile, the P/LP variants in the known PD-related genes might present other phenotypes rather than the characterized PD symptoms. For example, *LRRK2* variant carriers can present with PSP phenotype^46^, *PRKN* variant carriers with DRD, *ATP13A2*/*PLA2G6*/*FBXO7*/*SYNJ1*/*VPS13C* variant carriers with atypical Parkinsonian syndromes^11^. These confusions raise the question of the nomenclature of genetic movement disorders which has been discussed for a long time^47–49^. Thus in 2016, the international Parkinson and movement disorder society task force recommended a 2-axis nomenclature system with the phenotype followed by the gene, and divided the hereditary parkinsonism-related genes into 3 categories based on phenotype besides parkinsonism. The recommendation was updated in 2022^39,47^, in which, most mutated genes found in the study were included in the hereditary parkinsonism (*DCTN1* in atypical parkinsonism or complex phenotypes; *GCH1, PLA2G6*, and *TH* in combined phenotypes; *GBA*, *ATXN2* and *MAPT* in disorders that usually present with other phenotypes but can have predominant parkinsonism) except for *PSEN1* and *ATXN3*. Our work might improve the understanding of nomenclature system and offer further clues to the system.

This study has some limitations. Firstly, we did not apply whole-exome sequencing (WES) and dynamic variant testing of SCA to all the patients which might bias the frequency of causative genes in other diseases in the cohort. Secondly, a PET for dopamine transporter was not available in some patients carrying causative genes of other diseases. A follow-up study was important to these patients. Thirdly, this study was conducted in a single center of tertiary hospital which may inevitably result in bias, thus more multicenter studies are necessary in the future to verify our findings.

In conclusion, we investigated the genetic characteristics of a group of clinically diagnosed PD patients. This study demonstrated the importance of genetic testing in the diagnosis in clinically diagnosed PD patients, especially in the early-onset patients or patients with family history. It provided some clues to the nomenclature of genetic movement disorders. This will be crucial in grouping the patients in clinical trials and developing the treatment for the etiology.

## Methods

### Participants

**Fig. 3:**
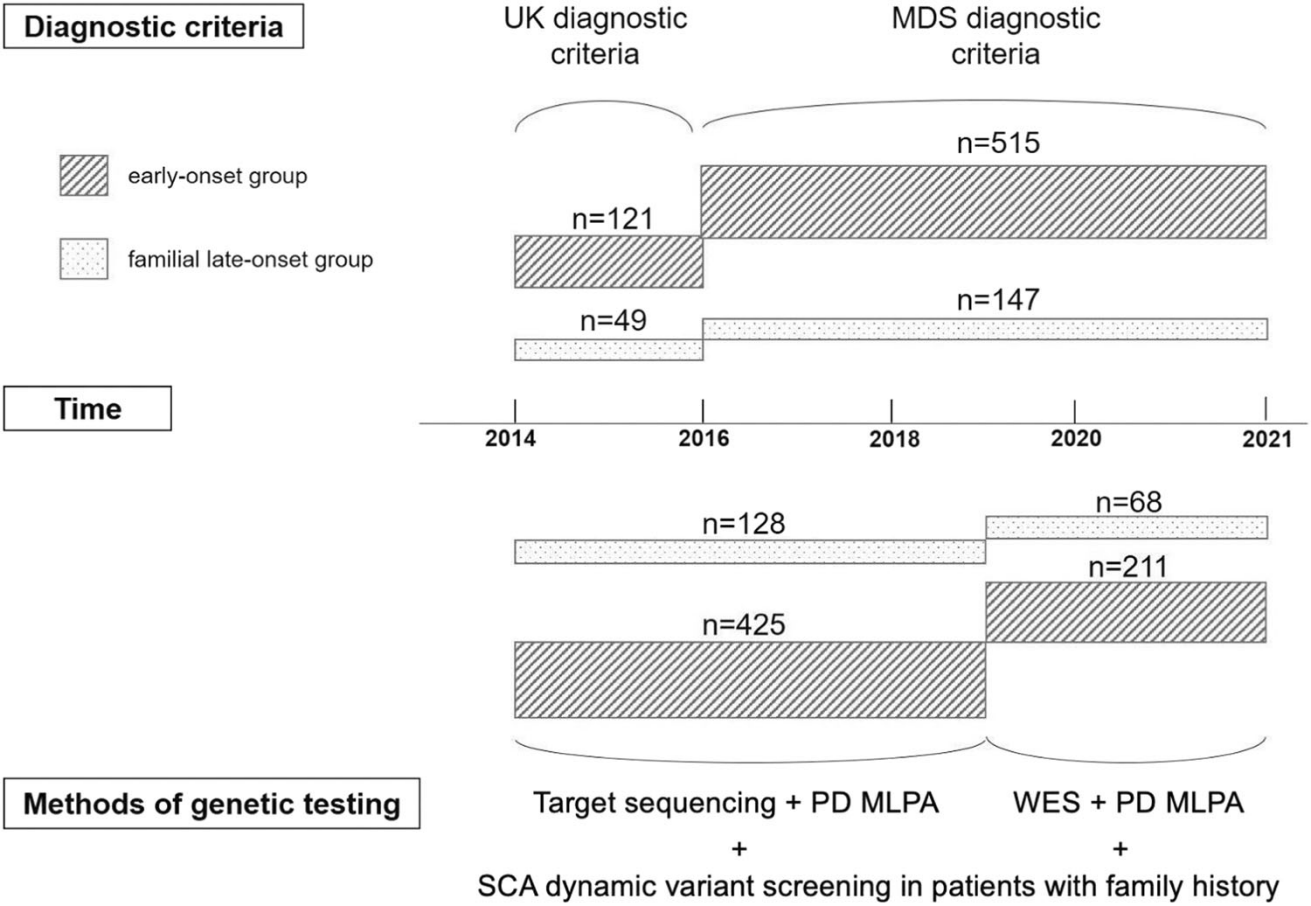
Procedure of the study. The upper part of the diagram indicated the diagnostic criteria used in patients of early-onset group and familial late-onset group clinically diagnosed as PD according to the time line. The bottom part of the diagram indicated the genetic testing carried out in the patients of different groups according to the time line. MDS diagnostic criteria: 2015 Movement Disorder Society Clinical Diagnostic Criteria for Parkinson’s disease, MLPA multiplex ligation-dependent probe amplification, PD Parkinson’s disease, SCA spinocerebellar ataxia, UK diagnostic criteria: The United Kingdom Parkinson’s disease Society Brain Bank Clinical Diagnostic Criteria, WES whole-exome sequencing.

Patients meeting the following criteria from February 2014 to December 2020 were investigated retrospectively: (1) a diagnosis of PD at the initial clinical visit; (2) AAO < 50, or AAO ≥ 50 and with family history of PD (defined as having at least one other affected relative in the family); and (3) consent to genetic testing related to PD. The diagnostic criteria used for PD were the United Kingdom PD Society Brain Bank Clinical Diagnostic Criteria^50^ (for patients recruited before 2016) or the 2015 Movement Disorder Society Clinical Diagnostic Criteria for PD^51^ (for patients recruited from 2016 on) (Fig. 3). The affected relatives were not included in the cohort.

Eight hundred and thirty-two patients were included. They were divided into “early-onset group” (AAO < 50, *n* = 636) and “familial late-onset group” (AAO ≥ 50 and with family history of PD, *n* = 196).

The study was approved by the Institutional Review Board of Huashan Hospital and the China human genetic resources management office. Written informed consent was obtained from all study participants.

### Clinical assessments of the patients initially diagnosed with PD

The clinical assessments were performed through a face-to-face interview with all patients. Baseline data were collected including demographic profiles (age, sex, education), disease history, family history, clinical signs, comorbidities, medications, and neurological examination results. An established method was used to calculate the LEDD^52^.

The Hoehn and Yahr (H&Y) scale and UPDRS motor examination (items 18–31) were conducted during the off-medication state, defined as withdrawal of anti-PD medications for at least 12 hours, except in those who could not tolerate it (*n* = 9). A battery of neuropsychological tests was performed to assess cognitive function and affected subdomains, as indicated in our previous study^53^. The non-motor symptoms were investigated by Epworth sleepiness scale (ESS), rapid eye movement sleep behavior disorder screening questionnaire (RBDSQ), Beck Depression Inventory (BDI), NMSQ, 39 item Parkinson’s disease questionnaire (PDQ39), and SSST-12, as indicated in our previous study^53^.

### Genetic testing and variants screening

Genomic deoxyribonucleic acid (DNA) samples were extracted from peripheral blood leukocytes of the probands and family members if necessary (Qiagen, Germany). The patients enrolled before January 2019 had genetic testing by target sequencing of a panel containing 116 movement-disorder-related genes (Supplementary Table 6), including 22 known PD-related genes (*ATP13A2*, *CHCHD2*, *DJ-1*, *DNAJC13*, *DNAJC6*, *EIF4G1*, *FBXO7*, *GBA*, *GIGYF2*, *HTRA2*, *LRRK2*, *PINK1*, *PLA2G6*, *POLG*, *PRKN*, *RAB39B*, *SNCA*, *SYNJ1*, *TMEM230, UCHL1*, *VPS13C*, *VPS35*)^3,4,54^, while the patients enrolled after that had genetic testing by WES (Fig. 1).

For target sequencing, genomic DNA was fragmented into 150–200 bp length by sonication. The DNA fragments were processed by end-repairing and enriched by a panel capturing the coding exons and corresponding flanking regions of 116 genes related to movement disorders (Supplementary Table 6). Paired-end sequencing was performed on lllumina HiSeq2000 platform to provide a mean read depth of over 100X and a coverage of at least 20X in more than 95% of targeted areas. Raw data was processed by the Illumina pipeline (version 1.3.4) for image analysis, error estimation, base calling and generating the primary sequence data. Variant calling was performed using Genome Analysis Toolkit (GATK) “Best Practices” workflow^55,56^. Briefly, after the removal of 3′-/5′- adapters and low-quality reads for the quality control, the clean reads were aligned to the human reference genome (Genome Reference Consortium Human Build 37 (GRCh37)/ human genome, version 19 (hg19)) using Burrow-Wheeler Aligner (BWA) (version: dynamic update with the time, http://bio-bwa.sourceforge.net) with default parameter settings. Polymerase chain reaction (PCR) duplicates were removed by Picard (version: dynamic update with the time, http://picard.sourceforge.net). Then we followed GATK (version: dynamic update with the time, https://software.broadinstitute.org/gatk/) standard pipelines to call germline variants.

As for WES, DNA libraries were prepared with KAPA Library Preparation Kit (Kapa Biosystems, KR0453) following the manufacturer’s instructions and DNA libraries were sequenced on the Illumina Novaseq platform with 200-bp paired-end mode. Similarly, we used GATK “Best Practices” workflow for germline variant calling, as previously described for target sequencing.

After variant calling steps above, all variants were annotated with ANNOtate VARiation (ANNOVAR) (https://annovar.openbioinformatics.org/en/latest/) tool^57^, and were further filtered with additional considerations from allele frequencies, variant damage predictions, and related phenotypes of corresponding genes. More specifically, variants with frequencies >1% were filtered according to 1000 Genomes Project (http://www.internationalgenome.org/data), Exome Sequencing Project v. 6500 (ESP6500) (evs.gs.washington.edu/EVS/), Exome Aggregation Consortium (ExAC) (EXac.broadinstitute.org/) and our inhouse database. Then, damaging missense variants were predicted by Sorting Intolerant From Tolerant (SIFT)^58^ (sift.bii.a-star.edu.sg/), Polymorphism Phenotyping v2 (PolyPhen-2)^59^ (genetics.bwh.harvard.edu/pph2/), and MutationTaster^60^ (www.mutationtaster.org/). The synonymous variants were excluded, and predicted damaging variants were passed as candidate variants. Besides, the phenotypes of the screened genes were compared with the clinical manifestations of the proband, and inherited modes were also taken into consideration in order to further exclude irrelevant genes.

The copy number variants (CNVs) in the 116 genes were analyzed by a bioinformatic tool of CNVkit^61^. The suspected CNV would be confirmed by real-time PCR.

The candidate variants with unknown pathogenicity were rated according to the American College of Medical Genetics (ACMG) guidelines^62^.

All the patients had multiplex ligation-dependent probe amplification (MLPA) of 8 PD-related genes (*ATP13A2*, *GCH1*, *LRRK2*, *PARK7*, *PINK1*, *PRKN*, *SNCA and UCHL1*) to investigate the CNVs. MLPA was performed using a SALSA MLPA Probemix P051-D1/P052-D2 Parkinson kit (MRC-Holland, the Netherland) according to the standard protocols provided by the manufacturer.

The candidate variants and any CNVs detected were further tested in the proband’s parents, other affected or unaffected family members for segregation and co-segregation by Sanger sequencing, real-time PCR or MLPA respectively, if necessary and possible, during which the homozygous or compound heterozygous status of the variants in genes with autosomal recessive inheritance would be confirmed.

The testing of dynamic variants was applied to the patients with family history, including SCA type 1, 2, 3, 6, 7, 8, 10, 12, 17 and Dentatorubral-pallidoluysian atrophy (DRPLA) by triplet repeat primed polymerase chain reaction (TP-PCR) and capillary electrophoresis techniques.

### The quality of the sequencing

As for the panel, the average sequencing depth of the target region was 393.65× and the mean percentage of the target region covered at least 20× was 99.35%. A total of 2153 regions were sequenced; 14 regions (0.65%) had read depths below 20×, which were mainly in GC-rich areas.

As for the WES, 251779 regions were sequenced. The average sequencing depth of the target region was 101.3x and the mean percentage of the target regions covered at least 20x was 80.6%. As for the 2235 regions in the 115 genes of interest, the average sequencing depth was 108.6x and the mean percentage covered at least 20x was 87.5%. The regions with sequencing depth below 20x in both target sequencing and WES were listed in Supplementary Table 7.

### Statistical analysis

All measurements were taken from distinct samples. The Shapiro–Wilk test was applied to test the normality. While categorical variables were demonstrated as frequencies (%), continuous variables were demonstrated as the median and IQR. The Chi-squared test, or Fisher’s exact test was used for comparing the categorical variables, and the Kruskal-Wallis test was used for comparing the continuous variables. Raw scores of neuropsychological tests were transformed into Z-scores as previously described^53^. The mean Z-scores of each domain from individual tests were computed. The generalized linear model (GLM) was used to evaluate the association between motor or non-motor scales and genetic status, which adjusted for age, sex, education, disease duration, and LEDD. Two-tailed *P* values were presented. Differences were considered statistically significant at *P* < 0.05. The data analysis was conducted by STATA 17.0 (StataCorp).

### Reporting summary

Further information on research design is available in the Nature Research Reporting Summary linked to this article.

## Supplementary information

Supplementary materials

Reporting Summary

## Data Availability

The cloud data sharing of the raw DNA sequencing data was not included in informed consent signed by the participants. The sequencing data can be available once the applicant’s institution and the research objective are verified by the corresponding author Jian Wang. The other data that support the findings in the study can be available from the request of the corresponding author Jian Wang. All the data should be applied for non-commercial purposes only, and sharing restrictions may be applied to sensitive data to preserve the participants’ privacy.

## Code availability

The bioinformatic analysis of the sequncing data was conducted as the previously reported sequencing analysis pipline, which is available at https://www.protocols.io/view/adbs-whole-exome-sequencing-wes-analysis-pipeline-8epv56rz6g1b/v1.
