# Supplementary material for: The genetic spectrum of a cohort of patients clinically diagnosed as Parkinson’s disease in mainland China
Source: NPJ Parkinsons Dis. 2023 May 17;9:76. doi: 10.1038/s41531-023-00518-9 (PMC10192372; doi:10.1038/s41531-023-00518-9)
Supplement: Supplementary file 1 — Supplementary materials [file 41531_2023_518_MOESM1_ESM.pdf]

Supplementary Materials

The genetic spectrum of a cohort of patients clinically diagnosed as Parkinson's disease in mainland China

Authors

Yi-Min Sun[1,a], M.D., Ph.D.; Xin-Yue Zhou[1,a], M.D.; Xiao-Niu Liang[1,a], Ph.D.; Jin-Ran Lin[2,a], B.S.; Yi-Dan Xu[1], M.D.; Chen Chen[1], M.D., Ph.D.; Si-Di Wei[1], M.D.; Qi-Si Chen[1], M.D.; Feng-Tao Liu[1], M.D., Ph.D.; Jue Zhao[1], M.D.; Yi-Lin Tang[1], M.D., Ph.D.; Bo Shen[1], M.D., Ph.D.; Lin-Hua Gan[1], Ph.D.; Boxun Lu[3], Ph.D.; Zheng-Tong Ding[1], M.D., Ph.D.; Yu An[2,*], Ph.D.; Jian-Jun Wu[1,*], M.D., Ph.D.; Jian Wang[1,*], M.D., Ph.D..

Authors' affiliations

1. Department of Neurology and National Research Center for Aging and Medicine & National Center for Neurological Disorders, State Key Laboratory of Medical Neurobiology, Huashan Hospital, Fudan University, Shanghai, China

2. Human Phenome Institute, Zhangjiang Fudan International Innovation Center, MOE Key Laboratory of Contemporary Anthropology, Fudan University, Shanghai, China

3. Neurology Department at Huashan Hospital, State Key Laboratory of Medical Neurobiology and MOE Frontiers Center for Brain Science, Institutes of Brain Science, School of Life Sciences, Fudan University, Shanghai, China

Supplementary Results

The mutational frequency of causative genes and the genotypes

All seven spinocerebellar ataxia type 2 (SCA2) patients carried a mild CAG-repeat expansion (35-37) and four of them had CAA interruptions [1], while the CAG-repeat numbers of five spinocerebellar ataxia type 3 (SCA3) patients were all in the fully penetrant range.

Among the 200 alleles with pathogenic/likely pathogenic (P/LP) *PRKN* variants, 123 (61.5%) had exon deletion/duplication/triplication, 74 (37%) had single nucleotide variants or small indels, and the other 3 (1.5%) carried both exon deletion/duplication and single nucleotide variants. The variants were commonly detected in exon 2-4 (47.5%).

The heterozygous *GBA* variants consisted of single nucleotide variants, small indels and splicing variants. *GBA* p.Leu483Pro variant was the most prevalent variant, accounting for 40.51% (32/79 alleles) of all the variants.

All the P/LP variants on 46 alleles of *PLA2G6* were single nucleotide variants. There is one hot single nucleotide variant of p.Asp331Tyr (c.G991T in exon 7), accounting for 17.4% (8/46 alleles) of all the variants.

We found 6 pedigrees carrying P/LP variants in *SNCA* including four with duplication and two with p.Ala53Thr, among which three pedigrees have been reported by us [2,3]. The length of genomic duplication in 4 pedigrees carrying *SNCA* was 139kb (f-LO-003), 5.4Mb (EO-114),1.58Mb (f-LO-017), and 160kb (EO-205), respectively, by array-based comparative genomic hybridization (aCGH) or whole genome sequencing (**Supplementary Figure 2**).

Two P/LP variants in *LRRK2* were found in the early onset group, including p.Arg1441Cys [4] in a patient without family history and p.Asn1437Asp [5] in a

patient with autosomal dominant family history of PD.

Two hundred healthy seniors recruited from the community epidemiologic investigations were applied target sequencing of 116 genes for the confirmation of the novel P/LP variants. None of the novel variants was found.

Figure legends

Supplementary Figure 1. Schematic representations of pathogenic/likely pathogenic variants identified in *CHCHD2, DCTN1, DJ-1, GBA, GCH1, HTRA2, LRRK2, MAPT, PINK1, PLA2G6, PRKN, PSEN1, SNCA, TH* and *VPS35* in this study.

Deletion/duplication of exons or splicing variants were labelled in the schematic diagram of exons. Variants in the coding areas were labelled in the schematic diagram of protein. Novel pathogenic/likely pathogenic variants were in red. The number on the top right of the variant indicate the times occurred in the cohort.

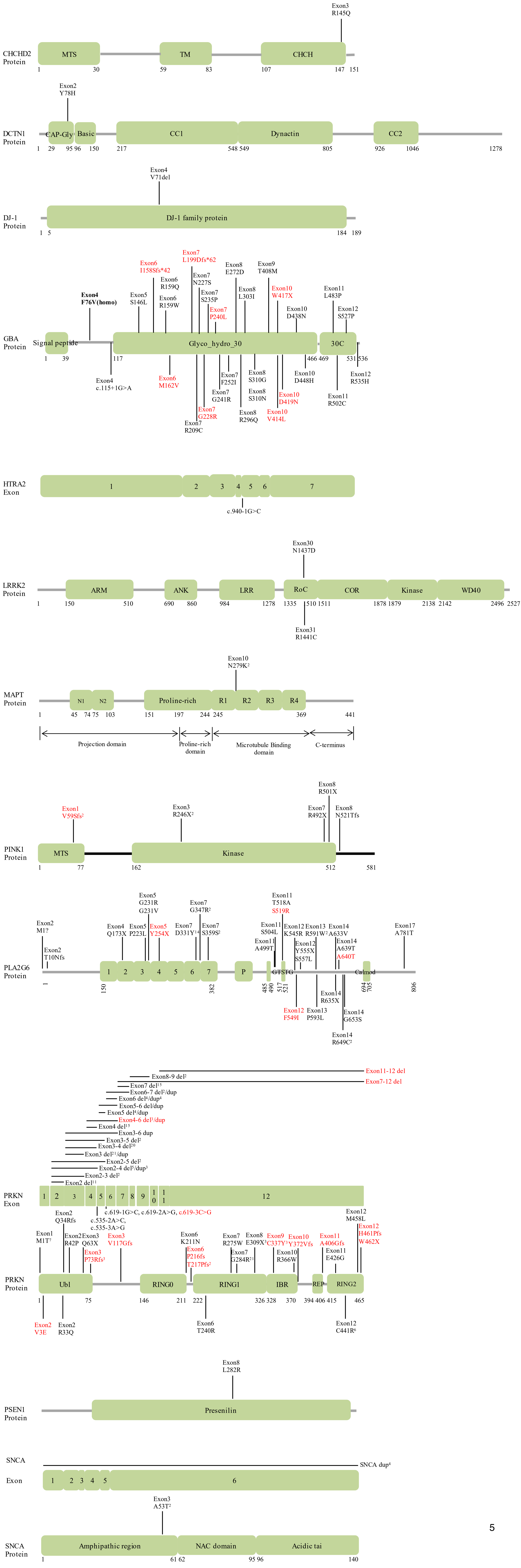

CHCHD2 Protein
MTS
TM
CHCH
Exon3 R145Q
1 30 59 83 107 147 151
DCTN1 Protein
Exon2 Y78H
CAP-Gly
Basic
CC1
Dynactin
CC2
1 29 95 96 150 217 548 549 805 926 1046 1278
DJ-1 Protein
Exon4 V71del
DJ-1 family protein
1 5 184 189
GBA Protein
Signal peptide
Glyco_hydro_30
30C
Exon4 F76V(homo)
Exon5 S146L
Exon6 1158Sfs*42
Exon6 R159Q
Exon6 R159W
Exon7 L199Dfs*62
Exon7 N227S
Exon7 S235P
Exon7 P240L
Exon8 E272D
Exon8 L303I
Exon9 T408M
Exon10 W417X
Exon10 D438N
Exon11 L483P
Exon12 S527P
Exon4 c.115+1G>A
Exon6 M162V
Exon7 R209C
Exon7 G228R
Exon7 G241R
Exon7 F252I
Exon8 S310G
Exon8 S310N
Exon8 R296Q
Exon10 D419N
Exon10 V414L
Exon10 D448H
Exon11 R502C
Exon12 R535H
1 39 117 466 469 531 536
HTRA2 Exon
1 2 3 4 5 6 7
c.940-1G>C
LRRK2 Protein
ARM
ANK
LRR
RoC
COR
Kinase
WD40
Exon30 N1437D
Exon31 R1441C
1 150 510 690 860 984 1278 1335 1510 1511 1878 1879 2138 2142 2496 2527
MAPT Protein
N1
N2
Proline-rich
R1 R2 R3 R4
Exon10 N279K2
1 45 74 75 103 151 197 244 245 369 441
Projection domain
Proline-rich domain
Microtubule Binding domain
C-terminus
PINK1 Protein
MTS
Kinase
Exon1 V59Sfs2
Exon3 R246X2
Exon8 R501X
Exon7 R492X
Exon8 N521Tfs
1 77 162 512 581
PLA2G6 Protein
1 2 3 4 5 6 7
P
GTSTG
Calmod
Exon2 M1?
Exon2 T10Nfs
Exon4 Q173X
Exon5 G231R
Exon5 G231V
Exon5 P223L
Exon5 Y254X
Exon7 G347R2
Exon7 D331Y14
Exon7 S359S2
Exon11 T518A
Exon11 S519R
Exon11 S504L
Exon11 A499T
Exon12 K545R
Exon12 Y555X
Exon12 S557L
Exon13 R591W2
Exon14 A633V
Exon14 A639T
Exon14 A640T
Exon17 A781T
Exon12 F549I
Exon13 P593L
Exon14 R635X
Exon14 G653S
Exon14 R649C2
1 150 382 485 490 517 521 694 705 806
PRKN Exon
1 2 3 4 5 6 7 8 9 10 11 12
Exon11-12 del
Exon7-12 del
Exon8-9 del2
Exon7 del15
Exon6-7 del2/dup
Exon6 del4/dup4
Exon5-6 del/dup
Exon5 del4/dup
Exon4-6 del3/dup
Exon4 del15
Exon3-6 dup
Exon3-5 del2
Exon3-4 del20
Exon3 del21/dup
Exon2-5 del2
Exon2-4 del3/dup3
Exon2-3 del2
Exon2 del11
c.619-1G>C, c.619-2A>G, c.619-3C>G
c.535-2A>C,
c.535-3A>G
PRKN Protein
Ubl
RING0
RING1
IBR
REP
RING2
Exon1 M1T7
Exon2 Q34Rfs
Exon2 R42P
Exon3 Q63X
Exon3 P73Rfs3
Exon3 V117Gfs
Exon6 K211N
Exon6 P216fs
Exon6 T217Pfs2
Exon7 R275W
Exon7 G284R21
Exon8 E309X3
Exon9 C337Y3
Exon10 Y372Vfs
Exon10 R366W
Exon11 A406Gfs
Exon11 E426G
Exon12 M458L
Exon12 H461Pfs
Exon12 W462X
Exon2 V3E
Exon2 R33Q
Exon6 T240R
Exon12 C441R6
1 75 146 211 222 326 328 370 394 406 415 465
PSEN1 Protein
Presenilin
Exon8 L282R
SNCA Exon
1 2 3 4 5 6
SNCA dup4
SNCA Protein
Amphipathic region
NAC domain
Acidic tai
Exon3 A53T2
1 61 62 95 96 140

Supplementary Figure 2. **The *SNCA* duplication identified in the study.**

(A.C) The duplication of exon 1-6 of *SNCA* was detected in f-LO-017 and EO-205 by multiplex ligation-dependent probe amplification (red square). (B) A 1.58Mb duplication (chr4: 90023715_91602310) (red arrow) contained 6 genes including *SNCA* detected by whole genome sequencing (f-LO-017). (D) A 160kb duplication (chr4: 90617242_90781117) (red arrow) contained 2 genes including *SNCA* by whole genome sequencing (EO-205). The results of *SNCA* duplication in other 2 probands were referred to our previous work [2].

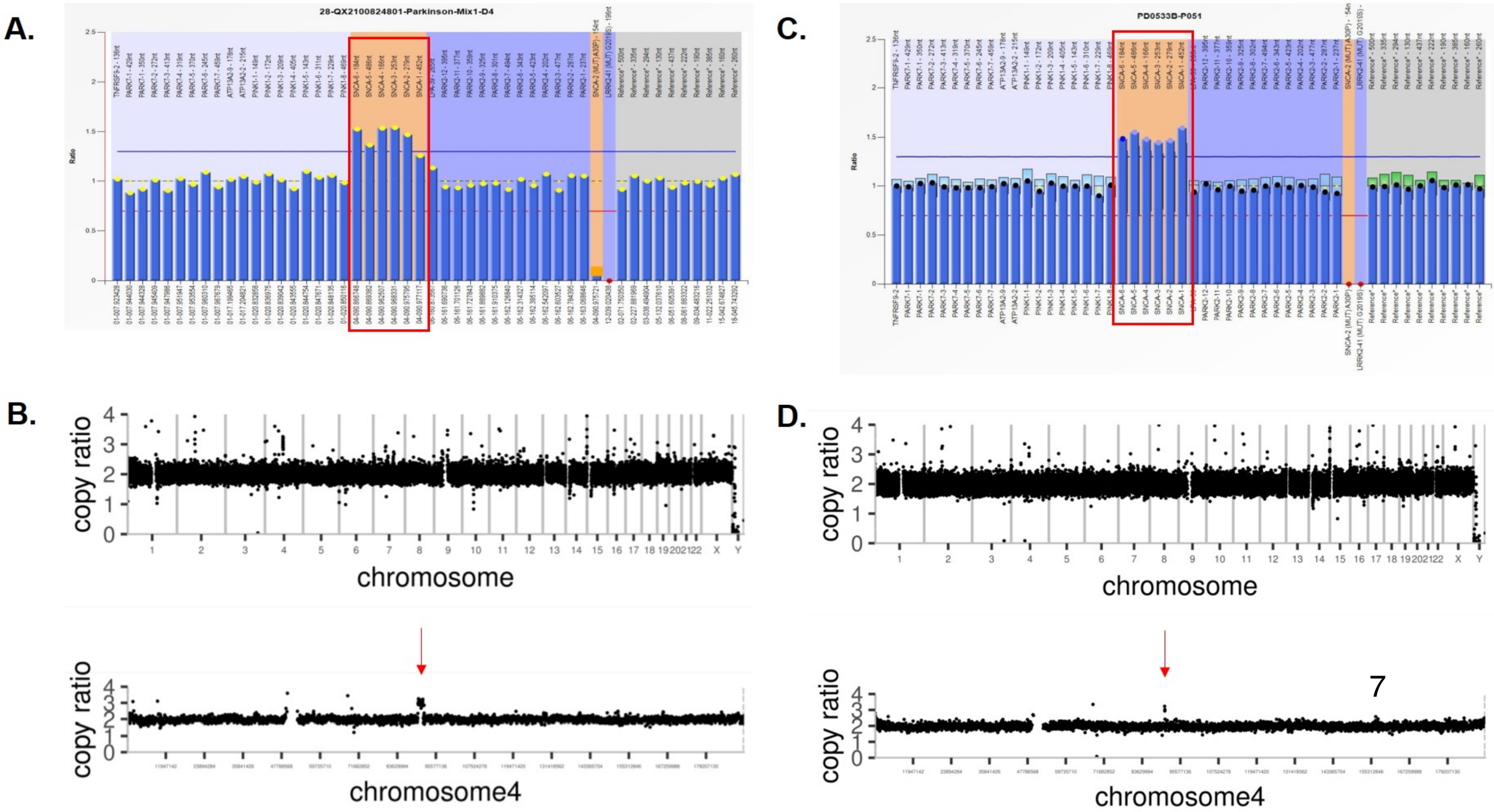
A.
28-QX2100824801-Parkinson-Mix1-D4
Ratio
B.
copy ratio
chromosome
copy ratio
chromosome4
C.
PD0533B-P051
Ratio
D.
copy ratio
chromosome
copy ratio
chromosome4

Reference

1 Mencacci, N. E. *et al.* Parkinson's disease in GTP cyclohydrolase 1 mutation carriers. *Brain : a journal of neurology* **137**, 2480-2492, doi:10.1093/brain/awu179 (2014).

2 Du, Y. J. *et al.* Clinical variability in Chinese families with Parkinson disease and SNCA duplication, including the shortest 139kb duplication. *Parkinsonism & related disorders* **68**, 60-62, doi:10.1016/j.parkreldis.2019.09.030 (2019).

3 Xiong, W. X. *et al.* The heterozygous A53T mutation in the alpha-synuclein gene in a Chinese Han patient with Parkinson disease: case report and literature review. *Journal of neurology* **263**, 1984-1992, doi:10.1007/s00415-016-8213-1 (2016).

4 Peng, F. *et al.* The heterozygous R1441C mutation of leucine-rich repeat kinase 2 gene in a Chinese patient with Parkinson disease: A five-year follow-up and literatures review. *Journal of the neurological sciences* **373**, 23-26, doi:10.1016/j.jns.2016.12.009 (2017).

5 Zhao, Y. *et al.* The role of genetics in Parkinson's disease: a large cohort study in Chinese mainland population. *Brain* **143**, 2220-2234, doi:10.1093/brain/awaa167 (2020).

Supplementary Table 1. The demographic and clinical features of the patients initially diagnosed with PD

Clinical features	Total (N=832)	Early-onset group (N=636)	Familial late-onset group (N=196)	p value
Sex, female (%)	352 (42.31)	256 (40.25)	96 (48.98)	**0.0306***
Education, years	11.00 (6.00)	12.00 (6.00)	9.00 (6.00)	**0.0001****
AAO, years	42.00 (14.00)	39.00 (12.00)	59.00 (10.00)	**0.0001****
Age at examination, years	48.00 (16.50)	44.00 (11.00)	63.50 (10.00)	**0.0001****
Disease duration, months	46.00 (65.00)	46.00 (67.00)	45.00 (57.00)	0.1371
LEDD, mg	400.00 (350.00)	410.00 (400.00)	400.0 (300.00)	0.8322
Motor				
UPDRS-part III, score (Med off)	29.00 (20.00)	28.00 (20.00)	29.00 (18.00)	0.7439
Non-motor manifestations				
BDI, score	12.00 (13.00)	12.00 (12.00)	13.00 (13.00)	0.7732
PDQ39, score	30.00 (36.00)	30.00 (37.00)	31.00 (35.00)	0.7106
NMSQ, score	10.00 (10.00)	10.00 (10.00)	12.00 (9.00)	**0.0406***
ESS, score	6.00 (6.00)	5.00 (5.00)	7.00 (8.00)	**0.0129***
RBDSQ, score	3.00 (3.00)	3.00 (3.00)	4.00 (5.00)	**0.0015****
SSST-12, score	6.00 (4.00)	7.00 (3.00)	5.00 (3.00)	**0.0001****
Cognitive characteristics				
Memory	0.06 (1.25)	0.20 (1.15)	-0.62 (1.21)	**0.0001****
Executive function	0.14 (0.82)	0.16 (0.75)	-0.12 (0.89)	**0.0002****
Language	0.05 (1.27)	0.13 (1.23)	-0.34 (1.25)	**0.0001****
Visuospatial function	0.17 (1.08)	0.23 (0.88)	-0.02 (1.21)	**0.0001****
Attention and working memory	0.15 (1.10)	0.26 (1.00)	-0.18 (1.29)	**0.0001****

Clinical features	Total (N=832)	Early-onset group (N=636)	Familial late-onset group (N=196)	p value
Raw neuropsychological tests scores				
MMSE, score	28.00 (1.00)	28.00 (1.00)	27.00 (5.00)	**0.0001****
Memory				
AVLT-delay recall, score	5.00 (4.00)	5.00 (3.00)	3.00 (4.00)	**0.0001****
AVLT-T, score	25.00 (14.50)	28.00 (14.00)	19.50 (12.00)	**0.0001****
CFT-delay recall, score	15.00 (11.00)	16.00 (10.00)	11.00 (11.50)	**0.0001****
Executive function				
CWT, second	73.00 (31.00)	71.00 (31.00)	79.00 (32.00)	**0.0014****
CWT right, score	47.00 (5.00)	48.00 (4.00)	45.00 (6.00)	**0.0001****
TMT-B, second	126.00 (73.00)	120.00 (63.00)	157.50 (86.50)	**0.0001****
Language				
BNT, score	24.00 (6.00)	24.00 (6.00)	22.00 (7.00)	**0.0001****
AFT, score	16.00 (7.00)	17.00 (7.00)	14.00 (6.00)	**0.0001****
Visuospatial function				
CFT, score	33.00 (5.00)	34.00 (3.00)	31.00 (11.00)	**0.0001****
CDT, score	20.00 (9.00)	20.00 (9.00)	21.00 (9.00)	0.2808
Attention and working memory				
SDMT, score	39.00 (11.00)	42.00 (19.00)	31.00 (20.00)	**0.0001****
TMT-A, second	53.50 (28.00)	52.00 (24.00)	67.00 (48.50)	**0.0001****

Abbreviations: AAO: age at onset; AFT: Animal Fluency Test; AVLT: Auditory Verbal Learning Test; AVLT-T: Auditory Verbal Learning Test total score; BDI: Beck Depression Inventory; BNT: Boston Naming Test; CDT: Clock Drawing Test; CFT: Rey-Osterrieth Complex Figure Test; CWT: Stroop Color-Word Test; ESS:

Epworth Sleepiness Score; LEDD: Levodopa equivalent dose daily; MMSE: Mini Mental State Examination; NMSQ: Non-Motor Symptoms Questionnaire; PDQ39: 39-item Parkinson's disease questionnaire; RBDSQ: Rapid-Eye-Movement Sleep Behavior Disorder Screening Questionnaire; SDMT: Symbol Digit Modalities Test; SSST-12: Sniffin' Sticks screening 12 test; TMT: Trail Making Test; UPDRS: Unified Parkinson's Disease Rating Scale.

The continuous data are presented as median (interquartile range, IQR), and the categorical data are presented as n (%).

Note: P Value: Comparison between the early-onset group and familial late-onset group.

** $p<0.01$, *$p<0.05$

Supplementary Table 2. Pathogenic/likely pathogenic variants detected in the study

Sample ID	Genetic testing	AAO	Sex	Het /Homo	Inheritance	Chr	Position	Gene	Nucleotide change	Amino acid change	Novel	ACMG	gnomAD_EAS	1000g_EAS	ExAC_EAS
EO-001	T+M	28	F	Homo	AR	chr22	38528924	*PLA2G6*	c.G991T	p.Asp331Tyr	-	-	-	-	0
EO-002	T+M	20	M	Het	AR	chr6	162206825	*PRKN*	c.G850C	p.Gly284Arg	-	-	-	-	-
EO-002	T+M	20	M	Het	AR	chr6	161771144	*PRKN*	c.G1385A	p.Trp462Ter	novel	PVS1+PM2+PM3=P	-	-	-
EO-003	T+M	23	F	Het	AR	chr6	6q26	*PRKN*	exon 7 del	-	-	-	-	-	-
EO-003	T+M	23	F	Het, de novo	AR	chr6	161969959	*PRKN*	c.G1010A	p.Cys337Tyr	novel	PM2+PM3+PM6+PP3=LP	-	-	-
EO-004	T+M	32	M	Het	AR	chr6	6q26	*PRKN*	exon 3-5 del	-	-	-	-	-	-
EO-004	T+M	32	M	Het	AR	chr6	162206825	*PRKN*	c.G850C	p.Gly284Arg	-	-	-	-	-
EO-005	T+M	5	M	Homo	AR	chr6	6q26	*PRKN*	exon 7 del	-	-	-	-	-	-
EO-006	T+M+S	34	M	Het	AR	chr6	162394349	*PRKN*	c.C719G	p.Thr240Arg	-	-	-	-	-
EO-006	T+M+S	34	M	Het	AR	chr6	6q26	*PRKN*	exon 3-4 del	-	-	-	-	-	-
EO-007	T+M	28	M	Het	AR	chr6	6q26	*PRKN*	exon 8-9 del	-	-	-	-	-	-
EO-007	T+M	28	M	Het	AR	chr6	6q26	*PRKN*	exon 3 del	-	-	-	-	-	-

Sample ID	Genetic testing	AAO	Sex	Het /Homo	Inheritance	Chr	Position	Gene	Nucleotide change	Amino acid change	Novel	ACMG	gnomAD_EAS	1000g_EAS	ExAC_EAS
EO-008	T+M+S	11	M	Homo	AR	chr6	6q26	*PRKN*	exon 3 del	-	-	-	-	-	-
EO-009	T+M	12	M	Homo	AR	chr6	6q26	*PRKN*	exon 3 del	-	-	-	-	-	-
EO-010	T+M	30	F	Het	AR	chr6	6q26	*PRKN*	exon 4 del	-	-	-	-	-	-
EO-010	T+M	30	F	Het	AR	chr6	6q26	*PRKN*	exon 3 dup	-	-	-	-	-	-
EO-011	T+M	21	M	Homo	AR	chr6	162864388	*PRKN*	c.G125C	p.Arg42Pro	-	-	-	-	-
EO-012	T+M+S	41	M	Het	AR	chr6	161990395	*PRKN*	c.G925T	p.Glu309Ter	novel	PVS1+PM2+PM3=P	-	-	-
EO-012	T+M+S	41	M	Het	AR	chr6	162206825	*PRKN*	c.G850C	p.Gly284Arg	-	-	-	-	-
EO-013	T+M+S	27	M	Het	AR	chr6	161990395	*PRKN*	c.G925T	p.Glu309Ter	novel	PVS1+PM2+PM3=P	-	-	-
EO-013	T+M+S	27	M	Het	AR	chr6	162206825	*PRKN*	c.G850C	p.Gly284Arg	-	-	-	-	-
EO-014	T+M	37	M	Homo	AR	chr6	6q26	*PRKN*	exon 4 del	-	-	-	-	-	-
EO-015	T+M	23	M	Het	AR	chr6	6q26	*PRKN*	exon 3 del	-	-	-	-	-	-
EO-015	T+M	23	M	Het	AR	chr6	162394452	*PRKN*	c.619-3C>G	-	-	-	-	-	-
EO-016	T+M	25	M	Het	AR	chr6	6q26	*PRKN*	exon 7 del	-	-	-	-	-	-
EO-016	T+M	25	M	Het	AR	chr6	162864415	*PRKN*	c.G98A & exon3 del	p.Arg33Gln & -	-	-	-	-	-

Sample ID	Genetic testing	AAO	Sex	Het /Homo	Inheritance	Chr	Position	Gene	Nucleotide change	Amino acid change	Novel	ACMG	gnomAD_EAS	1000g _EAS	ExAC_ EAS
EO-017	T+M	17	F	Homo	AR	chr6	162206825	*PRKN*	c.G850C	p.Gly284 Arg	-	-	-	-	-
EO-018	T+M	22	M	Het	AR	chr6	6q26	*PRKN*	exon 2 del	-	-	-	-	-	-
EO-018	T+M	22	M	Het	AR	chr6	162206825	*PRKN*	c.G850C	p.Gly284 Arg	-	-	-	-	-
EO-019	T+M	6	M	Homo	AR	chr1	8029417		c.205_207d el	p.Val71de l	novel	PM1+PM2+P M4+PP4=LP	-	-	-
EO-020	T+M	33	M	Homo	AR	chr6	6q26	*PRKN*	exon 4 del	-	-	-	-	-	-
EO-021	T+M	23	F	Het	AR	chr2 2	38528924	*PLA2G6*	c.G991T	p.Asp331 Tyr	-	-	-	-	0
EO-021	T+M	23	F	Het	AR	chr2 2	38115604	*PLA2G6*	c.G1957A	p.Gly653 Ser	-	-	-	-	-
EO-022	T+M	26	M	Het	AR	chr2 2	38511665	*PLA2G6*	c.C1903T	p.Arg635 Ter	-	-	-	-	-
EO-022	T+M	26	M	Het	AR	chr2 2	38528924	*PLA2G6*	c.G991T	p.Asp331 Tyr	-	-	-	-	0
EO-023	T+M	32	M	Homo	AR	chr6	162206825	*PRKN*	c.G850C	p.Gly284 Arg	-	-	-	-	-
EO-024	T+M	33	F	Het	AR	chr6	6q26	*PRKN*	exon 2 del	-	-	-	-	-	-
EO-024	T+M	33	F	Het	AR	chr6	6q26	*PRKN*	exon 4 del	-	-	-	-	-	-
EO-025	T+M	29	M	Homo	AR	chr6	6q26	*PRKN*	exon 3-4 del	-	-	-	-	-	-

Sample ID	Genetic testing	AAO	Sex	Het /Homo	Inheritance	Chr	Position	Gene	Nucleotide change	Amino acid change	Novel	ACMG	gnomAD_EAS	1000g_EAS	ExAC_EAS
EO-026	T+M	31	F	Het	AR	chr6	6q26	*PRKN*	exon 3 del	-	-	-	-	-	-
EO-026	T+M	31	F	Het	AR	chr6	6q26	*PRKN*	exon 3-4 del	-	-	-	-	-	-
EO-027	T+M	24	M	Het	AR	chr6	6q26	*PRKN*	exon 4-6 del	-	-	-	-	-	-
EO-027	T+M	24	M	Het	AR	chr6	162206825	*PRKN*	c.G850C	p.Gly284Arg	-	-	-	-	-
EO-028	T+M+S	26	M	Het	AR	chr22	38516863	*PLA2G6*	c.T1645A	p.Phe549Ile	novel	PM2+PM3+PP1+PP4=LP	-	-	-
EO-028	T+M+S	26	M	Het	AR	chr22	38519182	*PLA2G6*	c.C1511T	p.Ser504Leu	-	-	-	-	-
EO-029	T+M+S	24	F	Het	AR	chr6	161771208	*PRKN*	c.T1321C	p.Cys441Arg	-	-	-	-	-
EO-029	T+M+S	24	F	Het	AR	chr6	6q26	*PRKN*	exon 3 del	-	-	-	-	-	-
EO-030	T+M+S	27	F	Het	AR	chr6	161771208	*PRKN*	c.T1321C	p.Cys441Arg	-	-	-	-	-
EO-030	T+M+S	27	F	Het	AR	chr6	6q26	*PRKN*	exon 3 del	-	-	-	-	-	-
EO-031	T+M+S	32	F	Homo	AR	chr6	162206825	*PRKN*	c.G850C	p.Gly284Arg	-	-	-	-	-
EO-032	T+M	23	M	Homo	AR	chr6	6q26	*PRKN*	exon 7 del	-	-	-	-	-	-
EO-033	T+M+S	NA	M	Homo	AR	chr6	6q26	*PRKN*	exon 7 del	-	-	-	-	-	-

Sample ID	Genetic testing	AAO	Sex	Het /Homo	Inheritance	Chr	Position	Gene	Nucleotide change	Amino acid change	Novel	ACMG	gnomAD_EAS	1000g _EAS	ExAC_ EAS
EO-034	T+M	32	F	Het	AR	chr22	38536095	*PLA2G6*	c.G691C	p.Gly231 Arg	-	-	-	-	-
EO-034	T+M	32	F	Het	AR	chr22	38511670	*PLA2G6*	c.C1898T	p.Ala633 Val	novel	PM1+PM2+PM3+PP4=LP	-	-	-
EO-035	T+M+S	28	F	Het	AR	chr6	6q26	*PRKN*	exon 3 del	-	-	-	-	-	-
EO-035	T+M+S	28	F	Het	AR	chr6	161771147	*PRKN*	c.1381dup C	p.His461 Profs	-	PVS1+PM2+PM3=P	-	-	-
EO-036	T+M	24	F	Het	AR	chr6	6q26	*PRKN*	exon 2-5 del	-	-	-	-	-	-
EO-036	T+M	24	F	Het	AR	chr6	6q26	*PRKN*	exon 2 del	-	-	-	-	-	-
EO-037	T+M	19	F	Het	AR	chr6	6q26	*PRKN*	exon 7 del	-	-	-	-	-	-
EO-037	T+M	19	F	Het	AR	chr6	163148699	*PRKN*	c.T2C	p.Met1?	-	-	-	-	-
EO-038	T+M	30	F	Het	AR	chr6	6q26	*PRKN*	exon 3 del	-	-	-	-	-	-
EO-038	T+M	30	F	Het	AR	chr6	162864505	*PRKN*	c.T8A	p.Val3Glu	-	-	-	-	-
EO-039	T+M	24	M	Homo	AR	chr6	161990395	*PRKN*	c.G925T	p.Glu309 Ter	novel	PVS1+PM2=LP	-	-	-
EO-040	T+M	46	M	Het	AD	chr14	55369070	*GCH1*	c.C312A	p.Phe104 Leu	-	-	-	-	-
EO-041	T+M	15	M	Het	AR	chr6	6q26	*PRKN*	exon 2-4 del	-	-	-	-	-	-
EO-041	T+M	15	M	Het	AR	chr6	6q26	*PRKN*	exon 4 del	-	-	-	-	-	-
EO-042	T+M+S	37	M	Het	AR	chr6	6q26	*PRKN*	exon 6 del	-	-	-	-	-	-

Sample ID	Genetic testing	AAO	Sex	Het /Homo	Inheritance	Chr	Position	Gene	Nucleotide change	Amino acid change	Novel	ACMG	gnomAD_EAS	1000g_EAS	ExAC_EAS
EO-042	T+M+S	37	M	Het	AR	chr6	6q26	*PRKN*	exon 7 del	-	-	-	-	-	-
EO-043	T+M	33	F	Het	AR	chr6	6q26	*PRKN*	exon 4 del	-	-	-	-	-	-
EO-043	T+M	33	F	Het	AR	chr6	162683782	*PRKN*	c.C187T	p.Gln63Ter	novel	PVS1+PM2=LP	-	-	-
EO-044	T+M	40	F	Het	AR	chr6	6q26	*PRKN*	exon 3-4 del	-	-	-	-	-	-
EO-044	T+M	40	F	Het	AR	chr6	6q26	*PRKN*	exon 6-7	-	-	-	-	-	-
EO-045	T+M	26	M	Het, de novo	AR	chr6	6q26	*PRKN*	exon 5 del	-	-	-	-	-	-
EO-045	T+M	26	M	Het	AR	chr6	6q26	*PRKN*	exon 6 del	-	-	-	-	-	-
EO-046	T+M	12	M	Het	AR	chr6	162475209	*PRKN*	c.535-3A>G	-	novel	PM2+PM3+PP3+PP4=LP	-	-	-
EO-046	T+M	12	M	Het	AR	chr6	6q26	*PRKN*	exon 7 del	-	-	-	-	-	-
EO-047	T+M	21	M	Het	AR	chr6	6q26	*PRKN*	exon 3-5 del	-	-	-	-	-	-
EO-047	T+M	21	M	Het	AR	chr6	6q26	*PRKN*	exon 3-4 del	-	-	-	-	-	-
EO-048	T+M	29	F	Het	AR	chr6	6q26	*PRKN*	exon 2-4 del	-	-	-	-	-	-
EO-048	T+M	29	F	Het	AR	chr6	6q26	*PRKN*	exon 5-6 dup	-	-	-	-	-	-

Sample ID	Genetic testing	AAO	Sex	Het /Homo	Inheritance	Chr	Position	Gene	Nucleotide change	Amino acid change	Novel	ACMG	gnomAD_EAS	1000g_EAS	ExAC_EAS
EO-049	T+M+S	26	M	Het	AR	chr6	162683751	*PRKN*	c.218delC	p.Pro73Argfs*8	novel	PVS1+PM2+PM3+PP2=P	-	-	-
EO-049	T+M+S	26	M	Het	AR	chr6	6q26	*PRKN*	exon 2-4 dup	-	-	-	-	-	-
EO-050	T+M+S	29	M	Het	AR	chr6	162683751	*PRKN*	c.218delC	p.Pro73Argfs*8	novel	PVS1+PM2+PM3+PP2=P	-	-	-
EO-050	T+M+S	29	M	Het	AR	chr6	6q26	*PRKN*	exon 2-4 dup	-	-	-	-	-	-
EO-051	T+M	14	M	Het	AR	chr6	6q26	*PRKN*	exon 2-4 del	-	-	-	-	-	-
EO-051	T+M	14	M	Het	AR	chr6	6q26	*PRKN*	exon 6 del	-	-	-	-	-	-
EO-052	T+M	14	F	Het	AR	chr6	6q26	*PRKN*	exon 3-4 del	-	-	-	-	-	-
EO-052	T+M	14	F	Het	AR	chr6	161771208	*PRKN*	c.T1321C	p.Cys441Arg	-	-	-	-	-
EO-053	T+M	22	F	Homo	AR	chr6	6q26	*PRKN*	exon 6 dup	-	-	-	-	-	-
EO-054	T+M+S	19	F	Het	AR	chr6	6q26	*PRKN*	exon 2 del+exon 5 dup	-	-	-	-	-	-
EO-054	T+M+S	19	F	Het	AR	chr6	6q26	*PRKN*	exon 3 del	-	-	-	-	-	-
EO-055	T+M	49	F	Het	AD	chr14	55369143	*GCH1*	c.G239A	p.Ser80Asn	-	-	-	-	-

Sample ID	Genetic testing	AAO	Sex	Het /Homo	Inheritance	Chr	Position	Gene	Nucleotide change	Amino acid change	Novel	ACMG	gnomAD_EAS	1000g_EAS	ExAC_EAS
EO-056	T+M	32	F	Het	AR	chr6	6q26	*PRKN*	exon 3-4 del	-	-	-	-	-	-
EO-056	T+M	32	F	Het	AR	chr6	163148699	*PRKN*	c.T2C	p.Met1?	-	-	-	-	-
EO-057	T+M+S	33	M	Het	AR	chr6	6q26	*PRKN*	exon 3-4 del	-	-	-	-	-	-
EO-057	T+M+S	33	M	Het	AR	chr6	6q26	*PRKN*	exon 3 del	-	-	-	-	-	-
EO-058	T+M	32	F	Homo	AR	chr1	155209758	*GBA*	c.T226G	p.Phe76Val	-	-	-	-	-
EO-059	T+M+S	46	M	Het	AR	chr6	163148699	*PRKN*	c.T2C	p.Met1?	-	-	-	-	-
EO-059	T+M+S	46	M	Het	AR	chr6	6q26	*PRKN*	exon 3 del	-	-	-	-	-	-
EO-060	T+M	12	M	Homo	AR	chr6	162683618	*PRKN*	c.350_351del	p.Val117Glyfs*9	novel	PVS1+PM2=LP	-	-	-
EO-061	T+M	17	F	Homo	AR	chr1	20975710	*PINK1*	c.C1474T	p.Arg492Ter	-	-	0.0005985	0.001	0.0004638
EO-062	T+M	22	F	Het, de novo	AR	chr6	6q26	*PRKN*	exon 4 del	-	-	-	-	-	-
EO-062	T+M	22	F	Het	AR	chr6	6q26	*PRKN*	exon 6 del	-	-	-	-	-	-
EO-063	T+M	39	M	Homo	AR	chr6	6q26	*PRKN*	exon 2-3 del	-	-	-	-	-	-
EO-064	T+M	28	F	Homo	AR	chr6	162206825	*PRKN*	c.G850C	p.Gly284Arg	-	-	-	-	-
EO-065	T+M	23	F	Homo	AR	chr6	6q26	*PRKN*	exon 7 del	-	-	-	-	-	-

Sample ID	Genetic testing	AAO	Sex	Het /Homo	Inheritance	Chr	Position	Gene	Nucleotide change	Amino acid change	Novel	ACMG	gnomAD_EAS	1000g_EAS	ExAC_EAS
EO-066	T+M	26	M	Homo	AR	chr6	162206825	*PRKN*	c.G850C	p.Gly284Arg	-	-	-	-	-
EO-067	T+M	25	M	Het	AR	chr6	6q26	*PRKN*	exon 2-4 del	-	-	-	-	-	-
EO-067	T+M	25	M	Het, de novo	AR	chr6	6q26	*PRKN*	exon 3-4 del	-	-	-	-	-	-
EO-068	T+M	19	M	Het	AR	chr6	6q26	*PRKN*	exon 3-4 del	-	-	-	-	-	-
EO-068	T+M	19	M	Het	AR	chr6	6q26	*PRKN*	exon 7 del	-	-	-	-	-	-
EO-069	T+M	37	F	Het	AR	chr6	6q26	*PRKN*	exon 6 dup	-	-	-	-	-	-
EO-069	T+M	37	F	Het	AR	chr6	162394450	*PRKN*	c.619-1G>C	-	-	PVS1+PM2=LP	-	-	-
EO-070	T+M	30	F	Homo	AR	chr1	20960214	*PINK1*	c.173delG	p.Val59Serfs*48	novel	PVS1+PM2+PP4=P	-	-	-
EO-071	T+M	13	F	Homo	AR	chr6	162206852	*PRKN*	c.C823T	p.Arg275Trp	-	-	-	-	-
EO-072	T+M	44	F	Het	AD	chr12	40704236	*LRRK2*	c.C4321T	p.Arg1441Cys	-	-	0	.	0
EO-073	T+M	25	F	Homo	AR	chr6	162394435	*PRKN*	c.A633C	p.Lys211Asn	-	-	-	-	-
EO-074	T+M	31	M	Homo	AR	chr6	6q26	*PRKN*	exon 6-7 del	-	-	-	-	-	-

Sample ID	Genetic testing	AAO	Sex	Het /Homo	Inheri tance	Chr	Position	Gene	Nucleotide change	Amino acid change	Novel	ACMG	gnomAD_EAS	1000g _EAS	ExAC_ EAS
EO-075	T+M	36	F	Het	AD	chr1	155208006	*GBA*	c.A680G	p.Asn227 Ser	-	-	0	.	0.0001
EO-076	T+M	45	F	Het	AD	chr1	155210420	*GBA*	c.115+1G>A	-	-	-	0	0.001	0.0002
EO-077	T+M	49	F	Het	AD	chr1	155205043	*GBA*	c.T1448C	p.Leu483 Pro	-	-	0.0013	0.001	0.0013
EO-078	T+M	38	M	Het	AD	chr1	155210420	*GBA*	c.115+1G>A	-	-	-	0	0.001	0.0002
EO-079	T+M	37	F	Het	AD	chr1	155210420	*GBA*	c.115+1G>A	-	-	-	0	0.001	0.0002
EO-080	T+M	49	F	Het	AD	chr1	155210420	*GBA*	c.115+1G>A	-	-	-	0	0.001	0.0002
EO-081	T+M	29	F	Het	AD	chr1	155208090	*GBA*	c.595_596del	p.Leu199 Aspfs*62	novel	PVS1+PM1+ PM2=P	-	-	0
EO-082	T+M	42	M	Het	AD	chr1	155205043	*GBA*	c.T1448C	p.Leu483 Pro	-	-	0.0013	0.001	0.0013
EO-083	T+M	42	F	Het	AD	chr1	155210420	*GBA*	c.115+1G>A	-	-	-	0	0.001	0.0002
EO-084	T+M	44	M	Het	AD	chr1	155205043	*GBA*	c.T1448C	p.Leu483 Pro	-	-	0.0013	0.001	0.0013
EO-085	T+M	38	F	Het	AD	chr1	155205605	*GBA*	c.G1255A	p.Asp419 Asn	novel	PM1+PM2+PP3+PP4=LP	-	-	-

Sample ID	Genetic testing	AAO	Sex	Het /Homo	Inheritance	Chr	Position	Gene	Nucleotide change	Amino acid change	Novel	ACMG	gnomAD_EAS	1000g_EAS	ExAC_EAS
EO-086	T+M	43	F	Het	AD	chr1	155205043	*GBA*	c.T1448C	p.Leu483Pro	-	-	0.0013	0.001	0.0013
EO-087	T+M	34	M	Het	AD	chr1	155207224	*GBA*	c.C907A	p.Leu303Ile	-	-	-	-	-
EO-088	T+M	40	M	Het	AD	chr1	155208412	*GBA*	c.A484G	p.Met162Val	novel	PM1+PM2+PP3+PP4=LP	-	-	0
EO-089	T+M	44	M	Het	AD	chr1	155207932	*GBA*	c.T754A	p.Phe252Ile	-	-	0	.	0
EO-090	T+M	39	M	Het	AD	chr1	155208061	*GBA*	c.C625T	p.Arg209Cys	-	-	-	-	0
EO-091	T+M	39	F	Het	AD	chr1	155209424	*GBA*	c.C437T	p.Ser146Leu	-	-	-	-	0
EO-092	T+M	48	F	Het	AD	chr1	155205043	*GBA*	c.T1448C	p.Leu483Pro	-	-	0.0013	0.001	0.0013
EO-093	T+M	46	F	Het	AD	chr1	155207965	*GBA*	c.G721A	p.Gly241Arg	-	-	-	-	0
EO-094	T+M	45	F	Het	AD	chr1	155205518	*GBA*	c.G1342C	p.Asp448His	-	-	0.0006	.	0
EO-095	T+M	40	M	Het	AD	chr1	155207224	*GBA*	c.C907A	p.Leu303Ile	-	-	-	-	-
EO-096	T+M	33	F	Het	AD	chr1	155205043	*GBA*	c.T1448C	p.Leu483Pro	-	-	0.0013	0.001	0.0013

Sample ID	Genetic testing	AAO	Sex	Het /Homo	Inheritance	Chr	Position	Gene	Nucleotide change	Amino acid change	Novel	ACMG	gnomAD_EAS	1000g _EAS	ExAC_ EAS
EO-097	T+M	42	F	Het	AD	chr1	155207315	*GBA*	c.A816C	p.Glu272 Asp	-	-	-	-	-
EO-098	T+M	48	M	Het	AD	chr1	155204818	*GBA*	c.T1579C	p.Ser527 Pro	-	-	-	-	-
EO-099	T+M	45	M	Het	AD	chr1	155207202	*GBA*	c.G929A	p.Ser310 Asn	-	-	-	-	-
EO-100	T+M	39	F	Het	AD	chr1	155207203	*GBA*	c.A928G	p.Ser310 Gly	-	-	0.0006	.	0
EO-101	T+M	49	M	Het	AD	chr1	155210420	*GBA*	c.115+1G>A	-	-	-	0	0.001	0.0002
EO-102	T+M	49	M	Het	AD	chr1	155206101	*GBA*	c.T1159G	p.Trp387 Gly	-	-	-	-	0.0001
EO-103	T+M	47	F	Het	AD	chr1	155207965	*GBA*	c.G721A	p.Gly241 Arg	-	-	-	-	0
EO-104	T+M	35	F	Het	AD	chr1	155207203	*GBA*	c.A928G	p.Ser310 Gly	-	-	0.0006	.	0
EO-105	T+M	39	M	Het	AD	chr1	155208425	*GBA*	c.471delC	p.Ile158Serfs*42	novel	PVS1+PM1+PM2=P	-	-	-
EO-106	T+M	32	F	Het	AD	chr1	155205043	*GBA*	c.T1448C	p.Leu483 Pro	-	-	0.0013	0.001	0.0013
EO-107	T+M	45	F	Het	AD	chr1	155205043	*GBA*	c.T1448C	p.Leu483 Pro	-	-	0.0013	0.001	0.0013

Sample ID	Genetic testing	AAO	Sex	Het /Homo	Inheritance	Chr	Position	Gene	Nucleotide change	Amino acid change	Novel	ACMG	gnomAD_EAS	1000g_EAS	ExAC_EAS
EO-108	T+M	28	M	Het	AD	chr1	155205043	*GBA*	c.T1448C	p.Leu483Pro	-	-	0.0013	0.001	0.0013
EO-109	T+M+S	48	F	Het	AD	chr17	44087690	*MAPT*	c.837 T > G	p.Asn279Lys	-	-	-	-	-
EO-110	T+M+S	35	F	Het	AD	chr12	12q24.12	*ATXN2*	22/37	-	-	-	-	-	-
EO-111	T+M+S	49	F	Het	AR	chr6	162475208	*PRKN*	c.535-2A>C	-	novel	PVS1+PM2=LP	-	-	-
EO-111	T+M+S	49	F	Het	AR	chr6	161969959	*PRKN*	c.G1010A/ exon7 del	p.Cys337Tyr/-	novel/-	LP (rated previously)/-	-	-	-
EO-112	T+M+S	30	M	Homo	AR	chr6	162206825	*PRKN*	c.G850C	p.Gly284Arg	-	-	-	-	-
EO-113	T+M+S	22	M	Het	AD	chr4	90749300	*SNCA*	c.G157A	p.Ala53Thr	-	-	-	-	-
EO-114	T+M+S	38	M	Het	AD	chr4	4q22.1	*SNCA*	exon 1-6 dup	-	-	-	-	-	-
EO-115	T+M+S	28	F	Het	AD	chr14	14q32.12	*ATXN3*	27/72	-	-	-	-	-	-
EO-116	T+M+S	19	F	Het	AR	chr6	6q26	*PRKN*	exon 2 del	-	-	-	-	-	-
EO-116	T+M+S	19	F	Het	AR	chr6	6q26	*PRKN*	exon 3 del	-	-	-	-	-	-
EO-117	T+M+S	46	F	Het	AD	chr12	12q24.12	*ATXN2*	22/36	-	-	-	-	-	-

Sample ID	Genetic testing	AAO	Sex	Het /Homo	Inheritance	Chr	Position	Gene	Nucleotide change	Amino acid change	Novel	ACMG	gnomAD_EAS	1000g_EAS	ExAC_EAS
EO-118	T+M+S	23	F	Homo	AR	chr6	162206825	*PRKN*	c.G850C	p.Gly284Arg	-	-	-	-	-
EO-119	T+M+S	25	F	Het	AR	chr6	6q26	*PRKN*	exon 4 del	-	-	-	-	-	-
EO-119	T+M+S	25	F	Het	AR	chr6	161781128	*PRKN*	c.A1277G	p.Glu426Gly	novel	PM2+PM3+PP3+PP4=LP	-	-	-
EO-120	T+M+S	33	M	Het	AD	chr12	12q24.12	*ATXN2*	22/36	-	-	-	-	-	-
EO-121	T+M+S	35	M	Het	AD	chr14	14q32.12	*ATXN3*	14/67	-	-	-	-	-	-
EO-122	T+M+S	14	M	Het	AR	chr6	6q26	*PRKN*	exon 3 del	-	-	-	-	-	-
EO-122	T+M+S	14	M	Het	AR	chr6	162206825	*PRKN*	c.G850C	p.Gly284Arg	-	-	-	-	-
EO-123	T+M+S	26	F	Homo	AR	chr6	162206825	*PRKN*	c.G850C	p.Gly284Arg	-	-	-	-	-
EO-124	T+M+S	26	F	Het	AR	chr6	6q26	*PRKN*	exon 3-4 del	-	-	-	-	-	-
EO-124	T+M+S	26	F	Het	AR	chr6	162206825	*PRKN*	c.G850C	p.Gly284Arg	-	-	-	-	-
EO-125	T+M+S	21	M	Het	AR	chr6	6q26	*PRKN*	exon 2-4 triplication	-	novel	PVS1_Strong+PM2+PM3=LP	-	-	-

Sample ID	Genetic testing	AAO	Sex	Het /Homo	Inheritance	Chr	Position	Gene	Nucleotide change	Amino acid change	Novel	ACMG	gnomAD_EAS	1000g_EAS	ExAC_EAS
EO-125	T+M+S	21	M	Het	AR	chr6	6q26	*PRKN*	exon 2-5 del	-	-	-	-	-	-
EO-126	T+M+S	20	M	Het	AR	chr1	20976939	*PINK1*	c.C1501T	p.Arg501Ter	-	PVS1+PM2+PM3=P	-	-	-
EO-126	T+M+S	20	M	Het	AR	chr1	20966445	*PINK1*	c.C736T	p.Arg246Ter	-	-	0.0002188	.	0.0001444
EO-127	T+M+S	36	M	Het	AD	chr14	14q32.12	*ATXN3*	14/70	-	-	-	-	-	-
EO-128	T+M+S	35	F	Het	AR	chr6	6q26	*PRKN*	exon 3 del	-	-	-	-	-	-
EO-128	T+M+S	35	F	Het	AR	chr6	6q26	*PRKN*	exon 5 del	-	-	-	-	-	-
EO-129	T+M+S	32	M	Het	AD	chr12	12q24.12	*ATXN2*	22/37	-	-	-	-	-	-
EO-130	T+M+S	48	F	Het	AD	chr12	40703027	*LRRK2*	c.A4309G	p.Asn1437Asp	-	-	-	-	-
EO-131	T+M+S	39	M	Het	AD	chr16	46696364	*VPS35*	c.G1858A	p.Asp620Asn	-	-	-	-	-
EO-132	T+M+S	44	M	Homo	AR	chr6	162394420	*PRKN*	c.648delC	p.Thr217Profs*8	novel	PVS1+PM2=LP	-	-	-
EO-133	T+M+S	44	M	Het	AR	chr6	6q26	*PRKN*	exon 3-4 del	-	-	-	-	-	-
EO-133	T+M+S	44	M	Het	AR	chr6	161771208	*PRKN*	c.T1321C	p.Cys441Arg	-	-	-	-	-

Sample ID	Genetic testing	AAO	Sex	Het /Homo	Inheritance	Chr	Position	Gene	Nucleotide change	Amino acid change	Novel	ACMG	gnomAD_EAS	1000g_EAS	ExAC_EAS
EO-134	T+M+S	41	F	Het	AD	chr14	14q32.12	*ATXN3*	16/63	-	-	-	-	-	-
EO-135	T+M+S	44	F	Het	AD	chr17	44087690	*MAPT*	c.837 T > G	p.Asn279Lys	-	-	-	-	-
EO-136	T+M+S	44	F	Het	AD	chr1	155204987	*GBA*	c.C1504T	p.Arg502Cys	-	-	0	.	0
EO-137	T+M+S	45	F	Het	AD	chr1	155205518	*GBA*	c.G1342C	p.Asp448His	-	-	0.0006	.	0
EO-138	T+M+S	32	F	Het	AD	chr1	155205043	*GBA*	c.T1448C	p.Leu483Pro	-	-	0.0013	0.001	0.0013
EO-139	T+M+S	34	M	Het	AD	chr1	155207932	*GBA*	c.T754A	p.Phe252Ile	-	-	0	.	0
EO-140	T+M+S	45	M	Het	AD	chr1	155205043	*GBA*	c.T1448C	p.Leu483Pro	-	-	0.0013	0.001	0.0013
EO-141	T+M+S	31	M	Het	AD	chr1	155208004	*GBA*	c.G682A	p.Gly228Arg	novel	PM1+PM2+PP3+PP4=LP	-	-	-
EO-142	T+M+S	46	F	Het	AD	chr1	155208420	*GBA*	c.G476A	p.Arg159Gln	-	-	-	-	0
EO-143	T+M+S	45	M	Het	AD	chr1	155205043	*GBA*	c.T1448C	p.Leu483Pro	-	-	0.0013	0.001	0.0013
EO-144	T+M+S	40	M	Het	AD	chr1	155205518	*GBA*	c.G1342C	p.Asp448His	-	-	0.0006	.	0

Sample ID	Genetic testing	AAO	Sex	Het /Homo	Inheritance	Chr	Position	Gene	Nucleotide change	Amino acid change	Novel	ACMG	gnomAD_EAS	1000g_EAS	ExAC_EAS
EO-145	T+M+S	41	F	Het	AD	chr1	155205043	*GBA*	c.T1448C	p.Leu483Pro	-	-	0.0013	0.001	0.0013
EO-146	T+M+S	48	F	Het	AD	chr1	155205043	*GBA*	c.T1448C	p.Leu483Pro	-	-	0.0013	0.001	0.0013
EO-147	T+M+S	44	F	Het	AD	chr1	155207932	*GBA*	c.T754A	p.Phe252Ile	-	-	0	.	0
EO-148	T+M+S	45	M	Het	AD	chr1	155205043	*GBA*	c.T1448C	p.Leu483Pro	-	-	0.0013	0.001	0.0013
EO-149	T+M+S	42	F	Het	AD	chr1	155205043	*GBA*	c.T1448C	p.Leu483Pro	-	-	0.0013	0.001	0.0013
EO-150	T+M+S	48	F	Het	AD	chr1	155205043	*GBA*	c.T1448C	p.Leu483Pro	-	-	0.0013	0.001	0.0013
EO-151	W+M+S	24	F	Het	AR	chr6	161807881	*PRKN*	c.1112dupC	p.Tyr372Valfs*2	novel	PVS1+PM2+PM3=P	-	-	-
EO-151	W+M+S	24	F	Het	AR	chr6	6q26	*PRKN*	exon 4-6 dup	-	-	-	-	-	-
EO-152	W+M	29	M	Het	AR	chr6	6q26	*PRKN*	exon 3-4 del	-	-	-	-	-	-
EO-152	W+M	29	M	Het	AR	chr6	163148699	*PRKN*	c.T2C	p.Met1?	-	-	-	-	-
EO-153	W+M	32	F	Homo	AR	chr6	6q26	*PRKN*	exon 6-7 dup	-	-	-	-	-	-
EO-154	W+M	18	M	Homo	AR	chr6	6q26	*PRKN*	exon 4 del	-	-	-	-	-	-

Sample ID	Genetic testing	AAO	Sex	Het /Homo	Inheritance	Chr	Position	Gene	Nucleotide change	Amino acid change	Novel	ACMG	gnomAD_EAS	1000g _EAS	ExAC_ EAS
EO-155	W+M	28	M	Het	AR	chr6	6q26	*PRKN*	exon 2 del	-	-	-	-	-	-
EO-155	W+M	28	M	Het	AR	chr6	6q26	*PRKN*	exon 3-4 del	-	-	-	-	-	-
EO-156	W+M+S	30	M	Het	AR	chr6	6q26	*PRKN*	exon 4 del	-	-	-	-	-	-
EO-156	W+M+S	30	M	Het	AR	chr6	161771208	*PRKN*	c.T1321C	p.Cys441 Arg	-	-	-	-	-
EO-157	W+M+S	25	M	Het	AR	chr6	6q26	*PRKN*	exon 3-6 dup	-	-	-	-	-	-
EO-157	W+M+S	25	M	Het	AR	chr6	6q26	*PRKN*	exon 7 del	-	-	-	-	-	-
EO-158	W+M	32	M	Homo	AR	chr1	20977000	*PINK1*	c.1562_1563del	p.Asn521 Tfs*40	novel	PVS1+PM2= LP	-	-	-
EO-159	W+M	32	F	Het	AR	chr22	38536094	*PLA2G6*	c.G692T	p.Gly231 Val	-	-	-	-	0
EO-159	W+M	32	F	Het	AR	chr22	38528924	*PLA2G6*	c.G991T	p.Asp331 Tyr	-	-	-	-	0
EO-160	W+M+S	42	M	Het	AR	chr6	161969959	*PRKN*	c.G1010A/ exon7 del	p.Cys337 Tyr/-	novel/-	LP (rated previously)/-	-	-	-
EO-160	W+M+S	42	M	Hemi	AR	chr6	162206825	*PRKN*	c.G850C	p.Gly284 Arg	-	-	-	-	-
EO-161	W+M	22	M	Het	AR	chr22	38528876	*PLA2G6*	c.G1039A	p.Gly347 Arg	-	-	-	-	-

Sample ID	Genetic testing	AAO	Sex	Het /Homo	Inheritance	Chr	Position	Gene	Nucleotide change	Amino acid change	Novel	ACMG	gnomAD_EAS	1000g_EAS	ExAC_EAS
EO-161	W+M	22	M	Het	AR	chr22	38508248	*PLA2G6*	c.G2341A	p.Ala781Thr	-	-	-	-	-
EO-162	W+M+S	40	F	Het	AR	chr6	6q26	*PRKN*	exon 2-4 del	-	-	-	-	-	-
EO-162	W+M+S	40	F	Het	AR	chr6	163148699	*PRKN*	c.T2C	p.Met1?	-	-	-	-	-
EO-163	W+M+S	39	M	Het	AD	chr4	90749300	*SNCA*	c.G157A	p.Ala53Thr	-	-	-	-	-
EO-164	W+M	43	M	Het	AR	chr6	162394420	*PRKN*	c.648delC	p.Thr217Profs*8	novel	PVS1+PM2=LP	-	-	-
EO-164	W+M	43	M	Het	AR	chr6	6q26	*PRKN*	exon 3 del	-	-	-	-	-	-
EO-165	W+M	30	M	Het	AR	chr22	38512183	*PLA2G6*	c.C1778T	p.Pro593Leu	-	-	-	-	-
EO-165	W+M	30	M	Het	AR	chr22	38528924	*PLA2G6*	c.G991T	p.Asp331Tyr	-	-	-	-	0
EO-167	W+M	24	M	Het	AR	chr6	6q26	*PRKN*	exon 3-4 del	-	-	-	-	-	-
EO-167	W+M	24	M	Het	AR	chr6	6q26	*PRKN*	exon 5 del	-	-	-	-	-	-
EO-168	W+M+S	33	F	Het	AR	chr6	6q26	*PRKN*	exon 6 dup	-	-	-	-	-	-
EO-168	W+M+S	33	F	Het	AR	chr6	6q26	*PRKN*	exon 2 del	-	-	-	-	-	-
EO-169	W+M	23	F	Het	AR	chr6	162206825	*PRKN*	c.G850C	p.Gly284Arg	-	-	-	-	-
EO-169	W+M	23	F	Het	AR	chr6	163148699	*PRKN*	c.T2C	p.Met1?	-	-	-	-	-

Sample ID	Genetic testing	AAO	Sex	Het /Homo	Inheritance	Chr	Position	Gene	Nucleotide change	Amino acid change	Novel	ACMG	gnomAD_EAS	1000g _EAS	ExAC_ EAS
EO-170	W+M+S	38	M	Homo	AR	chr6	6q26	*PRKN*	exon 2-3 del	-	-	-	-	-	-
EO-171	W+M	47	M	Het	AR	chr1	20960214	*PINK1*	c.173delG	p.Val59Serfs*48	novel	PVS1+PM2+PP4=P	-	-	-
EO-171	W+M	47	M	Het	AR	chr1	20966445	*PINK1*	c.C736T	p.Arg246Ter	-	-	0.0002188	.	0.0001444
EO-172	W+M+S	17	M	Het	AR	chr22	38511650	*PLA2G6*	c.G1918A	p.Ala640Thr	novel	PM2+PM3+PP3+PP4+PP1=LP	-	-	-
EO-172	W+M+S	17	M	Het	AR	chr22	38512190	*PLA2G6*	c.C1771T	p.Arg591Trp	-	-	-	-	-
EO-173	W+M	34	M	Homo	AR	chr6	6q26	*PRKN*	exon 4 del		-	-	-	-	-
EO-174	W+M	22	M	Homo	AR	chr11	2189135	*TH*	c.G698A	p.Arg233His	-	-	-	-	-
EO-175	W+M	22	M	Homo	AR	chr6	6q26	*PRKN*	exon 4 del	-	-	-	-	-	-
EO-176	W+M	21	F	Het	AR	chr6	6q26	*PRKN*	exon 3-4 del	-	-	-	-	-	-
EO-176	W+M	21	F	Het	AR	chr6	6q26	*PRKN*	exon 5-6 del	-	-	-	-	-	-
EO-177	W+M	20	F	Het	AR	chr6	6q26	*PRKN*	exon 2 del	-	-	-	-	-	-
EO-177	W+M	20	F	Het	AR	chr6	6q26	*PRKN*	exon 7 del	-	-	-	-	-	-

Sample ID	Genetic testing	AAO	Sex	Het /Homo	Inheritance	Chr	Position	Gene	Nucleotide change	Amino acid change	Novel	ACMG	gnomAD_EAS	1000g_EAS	ExAC_EAS
EO-178	W+M+S	NA	M	Het	AR	chr6	6q26	*PRKN*	exon 3-4 del	-	-	-	-	-	-
EO-178	W+M+S	NA	M	Het	AR	chr6	162206825	*PRKN*	c.G850C	p.Gly284Arg	-	-	-	-	-
EO-179	W+M	34	M	Het	AR	chr22	38516874	*PLA2G6*	c.A1634G	p.Lys545Arg	-	-	-	-	-
EO-179	W+M	34	M	Het	AR	chr22	38528924	*PLA2G6*	c.G991T	p.Asp331Tyr	-	-	-	-	0
EO-180	W+M+S	37	M	Het	AR	chr6	6q26	*PRKN*	exon 4-6 del	-	-	-	-	-	-
EO-180	W+M+S	37	M	Het	AR	chr6	161807897	*PRKN*	c.C1096T	p.Arg366Trp	-	-	-	-	-
EO-181	W+M	40	F	Het	AD	chr1	155207932	*GBA*	c.T754A	p.Phe252Ile	-	-	0	.	0
EO-182	W+M	38	F	Het	AD	chr1	155207967	*GBA*	c.C719T	p.Pro240Leu	novel	PM1+PM2+PP3+PP4=LP	-	-	-
EO-183	W+M	44	M	Het	AD	chr1	155208006	*GBA*	c.A680G	p.Asn227Ser	-	-	0	.	0.0001
EO-184	W+M	35	F	Het	AD	chr1	155205043	*GBA*	c.T1448C	p.Leu483Pro	-	-	0.0013	0.001	0.0013
EO-185	W+M	39	M	Het	AD	chr1	155205609	*GBA*	c.G1251A	p.Trp417Ter	novel	PVS1+PM1+PM2=P	-	-	-

Sample ID	Genetic testing	AAO	Sex	Het /Homo	Inheritance	Chr	Position	Gene	Nucleotide change	Amino acid change	Novel	ACMG	gnomAD_EAS	1000g _EAS	ExAC_ EAS
EO-186	W+M	30	F	Het	AD	chr1	155205043	*GBA*	c.T1448C	p.Leu483 Pro	-	-	0.0013	0.001	0.0013
EO-187	W+M	26	M	Het	AD	chr1	155205043	*GBA*	c.T1448C	p.Leu483 Pro	-	-	0.0013	0.001	0.0013
EO-188	W+M	42	F	Het	AD	chr1	155205043	*GBA*	c.T1448C	p.Leu483 Pro	-	-	0.0013	0.001	0.0013
EO-189	W+M	32	F	Het	AD	chr1	155205043	*GBA*	c.T1448C	p.Leu483 Pro	-	-	0.0013	0.001	0.0013
EO-190	W+M	47	M	Het	AD	chr1	155205043	*GBA*	c.T1448C	p.Leu483 Pro	-	-	0.0013	0.001	0.0013
EO-191	W+M	42	M	Het	AD	chr1	155205043	*GBA*	c.T1448C	p.Leu483 Pro	-	-	0.0013	0.001	0.0013
EO-192	W+M	32	F	Het	AD	chr1	155205043	*GBA*	c.T1448C	p.Leu483 Pro	-	-	0.0013	0.001	0.0013
EO-193	W+M	31	M	Het	AD	chr1	155207244	*GBA*	c.G887A	p.Arg296 Gln	-	-	0	.	0
EO-194	W+M	46	F	Het	AD	chr1	155205518	*GBA*	c.G1342C	p.Asp448 His	-	-	0.0006	.	0
EO-195	W+M+S	47	M	Het	AR	chr6	6q26	*PRKN*	exon 3 del		-	-	-	-	-
EO-195	W+M+S	47	M	Het	AR	chr6	161771157	*PRKN*	c.A1372C	p.Met458 Leu	-	-	-	-	0

Sample ID	Genetic testing	AAO	Sex	Het /Homo	Inheritance	Chr	Position	Gene	Nucleotide change	Amino acid change	Novel	ACMG	gnomAD_EAS	1000g_EAS	ExAC_EAS
EO-196	W+M+S	41	M	Het	AD	chr14	14q32.12	*ATXN3*	14/63	-	-	-	-	-	-
EO-197	W+M+S	44	F	Het	AD	chr12	12q24.12	*ATXN2*	19/35	-	-	-	-	-	-
EO-198	W+M+S	45	M	Het	AD	chr7	56170571	*CHCHD2*	c.G434A	p.Arg145Gln	-	-	-	-	0
EO-199	W+M+S	29	M	Het	AR	chr6	161781188	*PRKN*	c.1216_1217insGCTCGTTGGGAAGCAG	p.Ala406Glyfs*168	novel	PVS1+PM2=LP	-	-	-
EO-199	W+M+S	29	M	Het	AR	chr6	162206825	*PRKN*	c.G850C	p.Gly284Arg	-	-	-	-	-
EO-200	W+M+S	29	M	Het	AR	chr6	162394451	*PRKN*	c.619-2A>G	-	novel	PVS1+PM2+PM3=P	-	-	-
EO-200	W+M+S	29	M	Het	AR	chr6	161771208	*PRKN*	c.T1321C	p.Cys441Arg	-	-	-	-	-
EO-201	W+M+S	33	M	Het	AR	chr6	6q26	*PRKN*	exon 3 del	-	-	-	-	-	-
EO-201	W+M+S	33	M	Het	AR	chr6	6q26	*PRKN*	exon 5 del	-	-	-	-	-	-
EO-202	W+M+S	25	M	Het	AR	chr22	38512190	*PLA2G6*	c.C1771T	p.Arg591Trp	-	-	-	-	-

Sample ID	Genetic testing	AAO	Sex	Het /Homo	Inheritance	Chr	Position	Gene	Nucleotide change	Amino acid change	Novel	ACMG	gnomAD_EAS	1000g_EAS	ExAC_EAS
EO-202	W+M+S	25	M	Het	AR	chr22	38511623	*PLA2G6*	c.C1945T	p.Arg649Cys	-	PM2+PM3+PP1+PP3+PP4=P	-	-	-
EO-203	W+M+S	16	F	Het	AR	chr6	163148699	*PRKN*	c.T2C	p.Met1?	-	-	-	-	-
EO-203	W+M+S	16	F	Het	AR	chr6	6q26	*PRKN*	exon 3 del	-	-	-	-	-	-
EO-204	W+M+S	16	F	Het	AR	chr6	6q26	*PRKN*	exon 3-4 del	-	-	-	-	-	-
EO-204	W+M+S	16	F	Het	AR	chr6	6q26	*PRKN*	exon 7 del	-	-	-	-	-	-
EO-205	W+M+S	49	F	Het	AD	chr4	4q22.1	*SNCA*	exon 1-6 dup	-	-	-	-	-	-
EO-206	W+M+S	23	F	Het	AR	chr22	38519141	*PLA2G6*	c.A1552G	p.Thr518Ala	novel	PM2+PM3+PP3+PP4=LP	-	-	-
EO-206	W+M+S	23	F	Het	AR	chr22	38528924	*PLA2G6*	c.G991T	p.Asp331Tyr	-	-	-	-	0
EO-207	W+M+S	47	M	Het	AD	chr1	155205043	*GBA*	c.T1448C	p.Leu483Pro	-	-	0.0013	0.001	0.0013
EO-208	W+M+S	43	F	Het	AD	chr1	155205043	*GBA*	c.T1448C	p.Leu483Pro	-	-	0.0013	0.001	0.0013
f-LO-001	T+M+S	53	F	Het	AD	chr2	74758693	*HTRA2*	c.940-1G>C	-	novel	PVS1+PM2=LP	0.002883	0.002	-
f-LO-002	T+M+S	50	F	Het	AD	chr14	73664814	*PSEN1*	c.T845G	p.Leu282Arg	-	-	-	-	-

Sample ID	Genetic testing	AAO	Sex	Het /Homo	Inheritance	Chr	Position	Gene	Nucleotide change	Amino acid change	Novel	ACMG	gnomAD_EAS	1000g_EAS	ExAC_EAS
f-LO-003	T+M+S	61	M	Het	AD	chr4	4q22.1	*SNCA*	exon 1-6 dup	-	-	-	-	-	-
f-LO-004	T+M+S	52	F	Het	AD	chr12	12q24.12	*ATXN2*	20/36	-	-	-	-	-	-
f-LO-005	T+M+S	55	F	Het	AD	chr12	12q24.12	*ATXN2*	22/35	-	-	-	-	-	-
f-LO-006	T+M+S	62	M	Het	AD	chr1	155204793	*GBA*	c.G1604A	p.Arg535His	-	-	0	.	0
f-LO-007	T+M+S	51	M	Het	AD	chr1	155205043	*GBA*	c.T1448C	p.Leu483Pro	-	-	0.0013	0.001	0.0013
f-LO-008	T+M+S	50	M	Het	AD	chr1	155205548	*GBA*	c.G1312A	p.Asp438Asn	-	-	-	-	-
f-LO-009	T+M+S	51	F	Het	AD	chr1	155207203	*GBA*	c.A928G	p.Ser310Gly	-	-	0.0006	.	0
f-LO-010	T+M+S	57	F	Het	AD	chr1	155205043	*GBA*	c.T1448C	p.Leu483Pro	-	-	0.0013	0.001	0.0013
f-LO-011	T+M+S	56	M	Het	AD	chr1	155205620	*GBA*	c.G1240C	p.Val414Leu	novel	PM1+PM2+PP3+PP4=LP	-	-	0
f-LO-012	T+M+S	52	M	Het	AD	chr1	155205518	*GBA*	c.G1342C	p.Asp448His	-	-	0.0006	.	0
f-LO-013	T+M+S	60	F	Het	AD	chr1	155205043	*GBA*	c.T1448C	p.Leu483Pro	-	-	0.0013	0.001	0.0013

Sample ID	Genetic testing	AAO	Sex	Het /Homo	Inheritance	Chr	Position	Gene	Nucleotide change	Amino acid change	Novel	ACMG	gnomAD_EAS	1000g_EAS	ExAC_EAS
f-LO-014	T+M+S	53	F	Het	AD	chr1	155205043	*GBA*	c.T1448C	p.Leu483Pro	-	-	0.0013	0.001	0.0013
f-LO-015	T+M+S	52	F	Het	AD	chr1	155208006	*GBA*	c.A680G	p.Asn227Ser	-	-	0	.	0.0001
f-LO-016	T+M+S	50	F	Het	AD	chr1	155205043	*GBA*	c.T1448C	p.Leu483Pro	-	-	0.0013	0.001	0.0013
f-LO-017	W+M+S	62	F	Het	AD	chr4	4q22.1	*SNCA*	exon 1-6 dup	-	-	-	-	-	-
f-LO-018	W+M+S	50	F	Het	AD	chr2	74605174	*DCTN1*	c.T232C	p.Tyr78His	-	-	-	-	-
f-LO-019	W+M+S	70	M	Het	AD	chr1	155207983	*GBA*	c.T703C	p.Ser235Pro	-	-	0	.	0
f-LO-020	W+M+S	60	M	Het	AD	chr1	155206037	*GBA*	c.C1223T	p.Thr408Met	-	reported as risk variants in PD	0	.	0.0001
f-LO-021	W+M+S	74	F	Het	AD	chr1	155208421	*GBA*	c.C475T	p.Arg159Trp	-	-	0	.	-

Abbreviations: AAO, age at onset; ACMG: the American College of Medical Genetics and Genomics AD, autosomal dominant model; A, atypical group; AR, autosomal recessive model; EAS, East Asian; EO, early-onset group; F, female; f-LO, familial late-onset group; LP, likely pathogenic; M, male; N, the variant is not novel; P, pathogenic. PD, Parkinson's disease; T+M: Target sequencing and multiplex ligation-dependent probe amplification of 8 genes related with

Parkinson’ s disease; T+M+S: Target sequencing, multiplex ligation-dependent probe amplification of 8 genes related with Parkinson’ s disease and testing of dynamic variants of spinocerebellar ataxias; W+M: Whole-exome sequencing and multiplex ligation-dependent probe amplification of 8 genes related with Parkinson’ s disease; W+M+S: Whole-exome sequencing, multiplex ligation-dependent probe amplification of 8 genes related with Parkinson’ s disease and testing of dynamic variants of spinocerebellar ataxias; - in ACMG column indicates the variant is previously reported as pathogenic; - in gnomAD EAS, 1000g EAS, ExAC-EAS indicates the variant is not reported in the database.

PVS, PM, PP in the ACMG column means different weight of pathogenic criterion as ‘very strong’, ‘moderate’ and ‘supporting’ according to ACMG guideline.

Supplementary Table 3. The neuropsychiatric assessments of the patients with pathogenic/likely pathogenic variants in *GBA*, *PRKN*, *PLA2G6* and genetic undefined patients in early-onset group.

Clinical features	GU-EOPD (N=429)	*PRKN* (N=100)	*GBA* (N=65)	*PLA2G6* (N=12)	P (patients with specific gene vs. GU-EOPD)		
					PRKN	*GBA*	*PLA2G6*
Sex, female (%)	160 (37.30)	41 (41.00)	36 (55.38)	5 (41.67)	0.4920	**0.0055****	0.7576
Education, years	12.00 (6.00)	12.00 (6.00)	11.00 (7.00)	12.50 (8.00)	0.9253	0.6765	0.6058
AAO, years	41.00 (9.00)	27.00 (11.00)	42.00 (8.00)	27.00 (8.00)	**0.0001****	0.4419	**0.0001****
Age at examination, years	45.00 (11.00)	37.00 (13.50)	45.00 (10.00)	30.00 (4.50)	**0.0001****	0.7449	**0.0001****
Disease duration, month	43.00 (60.00)	93.0 (123.50)	31.00 (54.00)	33.00 (39.00)	**0.0001****	**0.0314***	0.3928
LEDD, mg	450.00 (400.00)	350.00 (400.00)	475.00 (375.00)	350.00 (400.00)	**0.0034***	0.1294	0.8828
**Raw neuropsychological tests scores**							
**Memory**							
AVLT-T, score	28.00 (13.00)	29.00 (11.00)	27.50 (15.00)	20.50 (9.00)	0.1247	0.3446	**0.0290***[a]
AVLT-delay recall, score	5.00 (3.00)	6.00 (4.00)	5.00 (4.00)	2.00 (2.00)	0.1786	0.7955	**0.0175*** [a]
CFT-delay recall, score	16.00 (11.00)	16.00 (9.00)	14.50 (13.00)	7.50 (7.00)	0.9052	0.4938	**0.0046****[a]
**Executive function**							
CWT-time, second	72.00 (31.00)	63.00 (26.00)	76.00 (32.00)	95.00 (57.00)	**0.0034****	0.9435	0.0916[a]
CWT-right, score	48.00 (4.00)	49.00 (4.00)	47.00 (4.00)	44.00 (6.00)	**0.0116***	0.2160	0.0590[a]
TMT-B, second	125.50 (67.50)	99.00 (50.00)	107.50 (68.00)	128.00 (164.00)	**0.0001****[a]	0.0996	0.4214[a]
**Language**							
BNT, score	24.00 (5.50)	24.50 (4.50)	22.00 (6.50)	17.50 (11.00)	0.2308	0.1322	**0.0158*** [a]
AFT, score	17.00 (7.00)	19.50 (8.00)	16.00 (7.00)	12.00(11.00)	**0.0006****[a]	0.7737	0.3394

Clinical features	GU-EOPD (N=429)	*PRKN* (N=100)	*GBA* (N=65)	*PLA2G6* (N=12)	P (patients with specific gene vs. GU-EOPD)		
					PRKN	*GBA*	*PLA2G6*
**Visuospatial function**							
CFT, score	34.00 (3.00)	34.00 (3.00)	33.00 (4.50)	31.00 (17.00)	0.8831	0.1307 [a]	0.2764
CDT, score	21.00 (9.00)	21.00 (6.00)	18.00 (12.00)	15.00 (9.00)	0.9679	0.4479	**0.0131*[a]**
**Attention and working memory**							
SDMT, score	41.00 (18.00)	45.00 (18.00)	46.00 (23.00)	27.00 (12.00)	**0.0022****	0.1118	0.0519[a]
TMT-A, second	53.00 (24.00)	46.00 (20.00)	49.00 (27.00)	59.00 (66.00)	**0.0001**[a]**	0.1896	**0.0481*[a]**

Abbreviations: AAO: age at onset; AFT: Animal Fluency Test; AVLT: Auditory Verbal Learning Test; AVLT-T: Auditory Verbal Learning Test total score; BDI: Beck Depression Inventory; BNT: Boston Naming Test; CDT: Clock Drawing Test; CFT: Rey-Osterrieth Complex Figure Test; CWT: Stroop Color-Word Test; EOPD: early-onset PD; GU: genetically undefined; LEDD: Levodopa equivalent dose daily; SDMT: Symbol Digit Modalities Test; TMT: Trail Making Test.

The continuous data are presented as median (interquartile range, IQR).

Note: P: Comparison between the early-onset group with pathogenic/likely pathogenic variants in specific genes and GU-EOPD group. *P < 0.05, ** P <0.001, [a] significant after adjustment for age, gender, education, disease duration, and levodopa dose equivalents.

Supplementary Table 4. Clinical features of the patients carrying pathogenic/likely pathogenic variants in PD causative genes with patient number less than 10 in early-onset group or familial late-onset group

Clinical features	EO-198	EO-019	EO-072	EO-130	EO-131	f-LO-001	f-LO-017	f-LO-003	Reference
Variants	*CHCHD2* p.Arg145Gln het	*DJ-1* p.Val71del homo	*LRRK2* p.Arg1441Cys het	*LRRK2* p.Asn1437Asp het	*VPS35* p.Asp620Asn het	*HTRA2* c.940-1G>C het	*SNCA* Duplication het	*SNCA* Duplication het	
Family history	AD	Consanguineous marriage	Absent	AD	AD	AD	AD	AD	
AAO, years	45	6	44	48	39	53	62	61	
Disease duration, months	20	338	32	128	31	77	46	30	
UPDRS-Part III, score (Med off)	13	52	18	33	40	29 (med-on)	47	32	
Hoehn and Yahr stage	1	4	1	3	2	2	2	2	
**Non-motor manifestations**									
BDI, score	11	7	9	20↓	0	NA	33↓	19↓	[1]
PDQ39, score	17	74	26	64	2	15	26	10	
NMSQ, score	2	22	12	20	3	NA	24	7	
ESS, score	1	8↓	9↓	12↓	3	NA	10↓	6	[2]
RBDSQ, score	1	7↓	1	3	2	NA	4	1	[3]
SSST-12, score	6↓	9↓	5↓	7↓	6↓	NA	NA	NA	[4]
**Raw neuropsychological tests scores**									
MMSE, score	29	28	29	24↓	28	NA	19↓	25	[5]

Clinical features	EO-198	EO-019	EO-072	EO-130	EO-131	f-LO-001	f-LO-017	f-LO-003	Reference
Variants	*CHCHD2* p.Arg145Gln het	*DJ-1* p.Val71del homo	*LRRK2* p.Arg1441Cys het	*LRRK2* p.Asn1437Asp het	*VPS35* p.Asp620Asn het	*HTRA2* c.940-1G>C het	*SNCA* Duplication het	*SNCA* Duplication het	
**Memory**									
AVLT-delay recall, score	5	0↓	7	6	9	NA	NA	3	[6]
AVLT-T, score	24	11↓	36	23	50	NA	NA	16↓	[6]
CFT-delay recall, score	20	3	21	12	23	NA	17	7	
**Executive function**									
CWT time, second	91	108	65	50	63	NA	60	99	
CWT right, score	50	47	46	38	50	NA	48	46	
TMT-B, second	138	191↓	90	164	65	NA	282↓	136	[7]
**Language**									
BNT, score	23	28	25	13	23	NA	13	21	
AFT, score	23	8	23	12	13	NA	8	11	
**Visuospatial function**									
CFT, score	33	26	36	33	35	NA	17	29	
CDT, score	15	21	19	21	13	NA	23	22	
**Attention and working memory**									
SDMT, score	33	28	60	24	43	NA	21	26	
TMT-A, second	34	71	54	57	40	NA	130↓	59	[7]

Clinical features	EO-113	EO-114	EO-163	EO-205	EO-061	EO-070	EO-158	EO-126	EO-171	Reference
Variants	*SNCA* p.Ala53Thr het	*SNCA* Duplication het	*SNCA* p.Ala53Thr het	*SNCA* Duplication het	*PINK1* p.Arg492Ter homo	*PINK1* p.Val59Ser fs*48 homo	*PINK1* p.Asn521 Tfs*40 homo	*PINK1* p.Arg501Ter & p.Arg246Ter compound het	*PINK1* p.Arg246Ter & p.Val59Serfs*48 compound het	
Family history	AD	AD	AD	AD	Absent	Absent	Absent	AD	Absent	
AAO, years	22	38	39	49	17	30	32	20	47	
Disease duration, months	23	100	168	12	122	14	126	25	44	
UPDRS-Part III, score (Med off)	47	22	31	39	33	35	53	6	23 (Med-on)	
Hoehn and Yahr stage	3	3	2	2	3	2	3	1	2	
**Non-motor manifestations**										
BDI, score	18↓	20↓	12	10	34↓	4	16↓	2	NA	[1]
PDQ39, score	51	71	29	20	54	6	31	5	19	
NMSQ, score	10	18	13	11	14	0	3	5	NA	
ESS, score	4	22↓	7	7	9↓	4	3	7	NA	[2]
RBDSQ, score	1	12↓	13↓	4	8↓	3	6↓	2	NA	[3]
SSST-12, score	4↓	2↓	6↓	2↓	7↓	10	8	12	NA	[4]
**Raw neuropsychological tests scores**										
MMSE, score	29	30	27	27	21↓	29	27	30	NA	[5]
**Memory**										
AVLT-delay recall, score	6	4↓	8	6	3↓	7	6	7	NA	[6]

Clinical features	EO-113	EO-114	EO-163	EO-205	EO-061	EO-070	EO-158	EO-126	EO-171	Reference
Variants	*SNCA* p.Ala53Thr het	*SNCA* Duplication het	*SNCA* p.Ala53Thr het	*SNCA* Duplication het	*PINK1* p.Arg492Ter homo	*PINK1* p.Val59Ser fs*48 homo	*PINK1* p.Asn521 Tfs*40 homo	*PINK1* p.Arg501Ter & p.Arg246Ter compound het	*PINK1* p.Arg246Ter & p.Val59Serfs*48 compound het	
AVLT-T, score	32	27	36	18↓	13↓	33	28	36	NA	[6]
CFT-delay recall, score	32	5↓	14	7↓	10	32	17	34	NA	
**Executive function**										
CWT time, second	80	65	70	63	154↓	58	91	58	NA	
CWT right, score	49	50	48	40	22↓	50	35↓	49	NA	
TMT-B, second	115	94	156	291↓	182	82	94	55	NA	[7]
**Language**										
BNT, score	27	29	25	14↓	14↓	26	26	28	NA	
AFT, score	15	19	20	7↓	17	27	12	33	NA	
**Visuospatial function**										
CFT, score	36	30↓	35	23↓	29↓	36	31↓	30	NA	
CDT, score	28	17	15↓	20	10↓	21	23	30	NA	
**Attention and working memory**										
SDMT, score	49	61	24↓	28↓	37	59	48	65	NA	
TMT-A, second	49	42	80↓	140↓	182↓	35	51	18	NA	[7]

Abbreviations:

AVLT: Auditory Verbal Learning Test; AVLT-T: Auditory Verbal Learning Test total score; BDI: Beck Depression Inventory; BNT: Boston Naming Test; CDT: Clock Drawing Test; CFT: Rey-Osterrieth Complex Figure Test; CWT: Stroop Color-Word Test; EO: the early-onset group; ESS: Epworth Sleepiness Score; f-LO: the familial late-onset group; LEDD: Levodopa equivalent dose daily; MMSE: Mini Mental State Examination; NMSQ: Non-Motor Symptoms Questionnaire; PD: Parkinson’s disease; PDQ39: 39-item Parkinson’s disease questionnaire; RBDSQ: Rapid-Eye-Movement Sleep Behavior Disorder Screening Questionnaire; SDMT: Symbol Digit Modalities Test; SSST-12: Sniffin’ Sticks screening 12 test; TMT: Trail Making Test; UPDRS: Unified Parkinson’s Disease Rating Scale; NA: not available; ↓: impairment of non-motor symptoms, or cognition impairment of the cognitive test according to the references with cutoff score of each test by age and education level.

Reference:

1. Visser M, Leentjens AF, Marinus J, Stiggelbout AM, van Hilten JJ. Reliability and validity of the Beck depression inventory in patients with Parkinson's disease. Mov Disord. 2006 May;21(5):668-72.

2. Kurtis MM, Balestrino R, Rodriguez-Blazquez C, Forjaz MJ, Martinez-Martin P. A Review of Scales to Evaluate Sleep Disturbances in Movement Disorders. Front Neurol. 2018 May 29;9:369.

3. Stiasny-Kolster K, Mayer G, Schäfer S, Möller JC, Heinzel-Gutenbrunner M, Oertel WH. The REM sleep behavior disorder screening questionnaire--a new diagnostic instrument. Mov Disord. 2007 Dec;22(16):2386-93.

4. Hummel T, Konnerth CG, Rosenheim K, Kobal G. Screening of olfactory function with a four-minute odor identification test: reliability, normative data, and investigations in patients with olfactory loss. Ann Otol Rhinol Laryngol. 2001 Oct;110(10):976-81.

5. Zhang MY, Katzman R, Salmon D, Jin H, Cai GJ, Wang ZY, Qu GY, Grant I, Yu E, Levy P, et al. The prevalence of dementia and Alzheimer's disease in Shanghai, China: impact of age, gender, and education. Ann Neurol. 1990 Apr;27(4):428-37.

6. Guo Q, Zhao Q, Chen M, Ding D, Hong Z. A comparison study of mild cognitive impairment with 3 memory tests among Chinese individuals. Alzheimer Dis Assoc Disord. 2009 Jul-Sep;23(3):253-9.

7. LU Junchao, GUO Qi-hao, HONG Zhen, SHI Weixiong, LV Chuanzhen. Trail Making Test Used by Chinese Elderly Patients with Mild Cognitive Impairment and Mild Alzheimer' Dementia. CHINESE JOURNAL OF CLINICAL PSYCHOLOGY. 2006, 14(2): 118-120.

Supplementary Table 5. Clinical features of the patients carrying pathogenic/likely pathogenic variants in causative genes of other diseases initially diagnosed as PD

Clinical features	f-LO-018	EO-040	EO-055	EO-058	EO-135	EO-109	f-LO-002	EO-174	EO-110	f-LO-004
Variants	*DCTN1* p.Tyr78His het	*GCH1* p.Phe104Leu het	*GCH1* p.Ser80Asn het	*GBA* p.Phe76Val homo	*MAPT* p.Asn279Lys het	*MAPT* p.Asn279Lys het	*PSEN1* p.Leu282Arg het	*TH* p.Arg233His homo	*ATXN2* 22/37	*ATXN2* 20/36
Family history	AR	Negative	Negative	Consanguineous marriage	AD	AD	AD	Negative	AD	AD
AAO, years	50	46	49	32	44	48	50	22	35	52
Disease duration at 1st examination, months	36	10	28	79	6	29	62	96	71	60
Motor symptoms	Tremor, bradykinesia	Tremor, bradykinesia, rigidity	Tremor, bradykinesia	Tremor, bradykinesia, rigidity, postural instability	Tremor, bradykinesia, rigidity	Tremor, bradykinesia	Tremor, bradykinesia, rigidity, postural instability	Bradykinesia, rigidity	Tremor, bradykinesia, rigidity, postural instability	Tremor, bradykinesia, rigidity, postural instability
Motor complications	Fluctuation, dyskinesia	-	-	Fluctuation, dyskinesia, freezing gait	-	Fluctuation	Fluctuation	-	Fluctuation	-
Other features	-	Sleep benefit	Sleep benefit	Low Gcase level 3.02 (Normal range 6.56-55.1).	Normal eye movement	Normal eye movement	Urinary incontinence, cognitive impairment, visual	Sleep benefit, foot dystonia	RBD, sleep benefit	RBD, depression

							hallucination, compulsive behavior. Medical history of stroke and epilepsy.			
UPDRS-part III, score (Med off)	31 (Med-on)	11	9	34	28	35	72 (Med-on)	NA	8	27
H&Y stage	3	1	1	3	2	1.5	5	1	3	3
MMSE	28	27	27	24	28	30	5	28	27	29
Cerebral MRI at baseline	Mild atrophy in frontal lobe	Unremarkable	Unremarkable	Unremarkable	Mild atrophy in frontotemporal lobe	Mild atrophy in frontotemporal lobe	Hemorrhage lesion in the right occipital lobe	Unremarkable	Unremarkable	Unremarkable
CFT PET/CT	Decreased	NA	NA	NA	Decreased	Decreased	NA	NA	NA	Decreased
Follow-up	NA	3 years after onset, symptoms were stable (followed up by telephone)	4 years after onset, symptoms were stable	10 years after onset, typical PD symptoms were observed.	4 years after onset, PSP-like symptoms were observed.	3 years after onset, FTD symptoms were observed.	NA	10 years after onset, the symptoms progressed, but no new symptoms occurred	12 years after onset, the symptoms progressed, freezing gait and dyskinesia occurred, without signs of ataxia.	12 years after onset, the symptoms progressed, dyskinesia and blepharospasm was observed.

Clinical features	EO-117	EO-120	EO-197	f-LO-005	EO-129	EO-115	EO-196	EO-127	EO-134	EO-121
Variants	*ATXN2* 22/36	*ATXN2* 22/36	*ATXN2* 19/35	*ATXN2* 22/35	*ATXN2* 22/37	*ATXN3* 27/72	*ATXN3* 14/63	*ATXN3* 14/70	*ATXN3* 16/63	*ATXN3* 14/67
Family history	AD	AD	AD	AD	AD	AD	AD	AD	AD	AD
AAO range, years	46	33	44	55	32	28	41	36	41	35
Disease duration at 1st examination, months	85	57	13	98	13	9	149	20	54	85
Motor symptoms	Tremor, bradykinesia, postural instability	Tremor, bradykinesia, rigidity, postural instability	Rigidity, foot dystonia	Tremor, bradykinesia, rigidity, postural instability	Bradykinesia, rigidity	Tremor, bradykinesia	Tremor, bradykinesia, rigidity, postural instability	Tremor, bradykinesia, postural instability	Bradykinesia, rigidity, postural instability	Tremor, bradykinesia, rigidity, postural instability
Motor complications	Fluctuation	Freezing of gait	Freezing of gait	-	-	-	Fluctuation, freezing of gait	-	Fluctuation	Fluctuation
Other features	Sleep benefit, RBD, constipation	None	Sleep benefit, RBD, constipation, depression	Sleep benefit, RBD, constipation, depression, olfactory impairment	Sleep benefit	Sleep benefit, RBD	Olfactory impairment, depression, sexual dysfunction, dementia,	Sleep benefit	Sleep benefit, depression	Sleep benefit

							peripheral neuropathy			
UPDRS-part III, score (Med off)	35	18	12	27	11	23	NA	5	52	NA
H&Y stage	3	4	2	3	2	2	3	1	5	3
MMSE	12	28	28	29	30	30	NA	28	22	NA
Cerebral MRI at baseline	Unremarkable	Unremarkable	Unremarkable	Unremarkable	Unremarkable	Unremarkable	Unremarkable	Unremarkable	Unremarkable	Unremarkable
CFT PET/CT	Decreased	Decreased	NA	NA	NA	Decreased	NA	Decreased	Decreased	NA
Follow-up	15 years after onset, the symptoms progressed. Frequent falling occurred and mild nystagmus was observed.	13 years after onset, the symptoms progressed. Fluctuation, dyskinesia, postural instability, and spastic torticollis/upper limb dystonia occurred.	NA	14 years after onset, the symptoms progressed. Frequent falling occurred.	NA	4 years after onset, the symptoms progressed. No new symptoms occurred.	NA	NA	NA	NA

Abbreviations: AAO: age at onset; AD: autosomal dominant inherited mode; AR: autosomal recessive inherited mode; CFT PET/CT: [^{11}C]-2b-carbomethoxy-3b-(4-fluorophenyltropane) positron emission computed tomography; FTD, frontotemporal dementia; H&Y: Hoehn and Yahr; LEDD: levodopa equivalent dose daily; MMSE: Mini Mental State Examination; NA: not available; PD, Parkinson's disease; PSP, progressive supranuclear palsy; RBD, rapid eye movement sleep behavior disorder; UPDRS: Unified Parkinson's Disease Rating Scale; -: absent.

Supplementary Table 6. The targeted 116 movement disorder related genes in the study

Gene symbol	Gene name	Mode of inheritance	Subtype or disease	OMIM	Location
ADCY5	Adenylate Cyclase 5	AD	ADCY5 Dyskinesia	600293	3q21.1
AFG3L2	Afg3-Like Matrix AAA Peptidase, Subunit 2	AD	SCA28	604581	18p11.21
ANO10	Anoctamin 10	AR	Spinocerebellar ataxia, autosomal recessive 10	613726	3p22.1-p21.33
ANO3	Anoctamin 3	AD	DYT 24	610110	11p14.3-p14.2
APTX	Aprataxin	AR	Ataxia with Oculomotor Apraxia Type 1	606350	9p21.1
ATM	Atm Serine/Threonine Kinase	AR	Ataxia-telangiectasia	607585	11q22.3
ATP13A2	Atpase 13A2	AR	PARK9	610513	1p36.13
ATP1A3	Atpase, Na+/K+ TRANSPORTING, ALPHA-3 POLYPEPTIDE	AD	DYT 12	182350	19q13.2
ATP6AP2	Atpase, H+ TRANSPORTING, LYSOSOMAL, ACCESSORY PROTEIN 2	XLR	X linked intellectual disability with epilepsy and parkinsonism	300556	Xp11.4
ATP7B	Atpase, Cu(2+)-Transporting, Beta Polypeptide	AR	Wilson's disease	606882	13q14.3
CACNA1A	Calcium Channel, Voltage-Dependent, P/Q Type, Alpha-1a Subunit	AD	episodic ataxia 2/SCA6	601011	19p13.13
CACNA1G	Calcium Channel, Voltage-Dependent, T Type, Alpha-1g Subunit	AD	SCA42	604065	17q21.33
CACNB4	Calcium Channel, Voltage-Dependent, Beta-4 Subunit	AD	episodic ataxia 5	601949	2q23.3
CASK	Calcium/Calmodulin-Dependent Serine Protein Kinase	XLD	*CASK* related disorder	300172	Xp11.4
CCDC88C	Coiled-Coil Domain-Containing Protein 88c	AD	SCA40	611204	14q32.11-q32.12

CHCHD2	Coiled-Coil-Helix-Coiled-Coil-Helix Domain-Containing Protein 2	AD	PARK22	616244	7p11.2
CHMP2B	Charged Multivesicular Body Protein 2b	AD	FTD-ALS	609512	3p11.2
CIZ1	Cdkn1a-Interacting Zinc Finger Protein 1	AD	DYT 23	611420	9q34.11
COASY	Coenzyme A Synthase	AR	Neurodegeneration with brain iron accumulation 6	609855	17q21.2
COL6A3	Collagen, Type Vi, Alpha-3	AR	DYT 27	120250	2q37.3
COQ2	Coenzyme Q2, Polyprenyltransferase	AD, AR	Primary Coenzyme Q10 Deficiency; Multiple system atrophy, susceptibility to	609825	4q21.23
CP	Ceruloplasmin	AR	Hypoceruloplasminemia	117700	3q24-q25.1
CYP27A1	Cytochrome P450, Subfamily Xxviia, Polypeptide 1	AR	Cerebrotendinous Xanthomatosis	606530	2q35
DCTN1	Dynactin 1	AD	Perry syndrome	601143	2p13.1
DNAJC13	Dnaj/Hsp40 Homolog, Subfamily C, Member 13	AD	PARK21	614334	3q22.1
DNAJC6	Dnaj/Hsp40 Homolog, Subfamily C, Member 6	AR	PARK19	608375	1p31.3
DNMT1	Dna Methyltransferase 1	AD	Autosomal dominant cerebellar ataxia, deafness, and narcolepsy	126375	19p13.2
DRD2	Dopamine Receptor D2	AD?/AR?	myoclonus dystonia syndrome ?	126450	11q23.2
DRD3	Dopamine Receptor D3	AD	Essential tremor 1	126451	3q13.31
EIF4G1	Eukaryotic Translation Initiation Factor 4-Gamma, 1	AD	PARK18	600495	3q27.1
ELOVL4	Elongation Of Very Long Chain Fatty Acids-Like 4	AD	SCA34	605512	6q14.1
ELOVL5	Elongation Of Very Long Chain Fatty Acids-Like 5	AD	SCA38	611805	6p12.1
FA2H	Fatty Acid 2-Hydroxylase	AR	Spastic paraplegia 35	611026	16q23.1
FBXO7	F-Box Only Protein 7	AR	PARK15	605648	22q12.3

FGF14	Fibroblast Growth Factor 14	AD	SCA27	601515	13q33.1
FTL	Ferritin Light Chain	AD	Neuroferritinopathy	134790	19q13.33
FUS	Fused In Sarcoma	AD	essential tremor 4, FTD-ALS	137070	16p11.2
FXN	Frataxin	AR	Friedreich Ataxia	606829	9q21.11
GBA	Glucosidase, Beta, Acid	AR	Gaucher's disease	606463	1q22
GCH1	Gtp Cyclohydrolase I	AD	DYT 5a/14	600225	14q22.2
GIGYF2	Grb10-Interacting Gyf Protein 2	AD	PARK11	612003	2q37.1
GNAL	Guanine Nucleotide-Binding Protein	AD	DYT 25	139312	18p11.21
GRID2	Glutamate Receptor, Ionotropic, Delta 2	AR	*GRID2*-related spinocerebellar ataxia	602368	4q22.1-q22.2
GRN	Frontotemporal Lobar Degeneration With Tdp43 Inclusions	AD	FTD	607485	17q21.31
HPCA	Hippocalcin	AR	DYT 2	142622	1p35.1
HTRA2	Htra Serine Peptidase 2	AD	PARK13	606441	2p13.1
IFRD1	Interferon-Related Developmental Regulator 1	AD	SCA18	603502	7q31.1
ISG15	Ubiquitin-Like Modifier	AR	Immunodeficiency 38 with basal ganglia calcification	147571	1p36.33
ITPR1	Inositol 1,4,5-Triphosphate Receptor, Type 1	AD	SCA15/29	147265	3p26.1
KCNA1	Potassium Channel, Voltage-Gated, Shaker-Related Subfamily, Member 1	AD	episodic ataxia 1	176260	12p13.32
KCND3	Potassium Voltage-Gated Channel, Shal-Related Subfamily, Member 3	AD	SCA19	605411	1p13.2
KCTD17	Potassium Channel Tetramerization Domain-Containing Protein 17	AD	DYT 26	616386	22q12.3
LRRK2	Leucine-Rich Repeat Kinase 2	AD	PARK8	609007	12q12
MAPT	Microtubule-Associated Protein Tau	AD	FTD/CBS/PSP	157140	17q21.31

MCCC1	3-METHYLCROTONYL-Coa CARBOXYLASE 1	AR	3-Methylcrotonyl-CoA carboxylase 1 deficiency	609010	3q27.1
MECP2	METHYL-Cpg-BINDING PROTEIN 2	XLD	Rett syndrome	300005	Xq28
MR1	Major Histocompatibility Complex, Class I-Related	AD	DYT 8	600764	1q25.3
NKX2-1	Nk2 Homeobox 1	AD	hereditary benign chorea	600635	14q13.3
PANK2	Pantothenate Kinase 2	AR	neurodegeneration with brain iron accumulation 1	606157	20p13
PARK2 (PRKN)	Parkinson Disease 2	AR	PARK2	600116	6q26
PARK7 (DJ-1)	Parkinson Disease 7	AR	PARK7	602533	1p36.23
PDGFB	Platelet-Derived Growth Factor, Beta Polypeptide	AD	Idiopathic basal ganglia calcification 5	190040	22q13.1
PDGFRB	Platelet-Derived Growth Factor Receptor, Beta	AD	Idiopathic basal ganglia calcification 4	173410	5q32
PDYN	Prodynorphin	AD	SCA23	131340	20p13
PEX7	Peroxisome Biogenesis Factor 7	AR	Refsum disease	601757	6q23.3
PHYH	PHYTANOYL-Coa HYDROXYLASE	AR	Refsum disease	602026	10p13
PINK1	Pten-Induced Kinase 1	AR	PARK6	608309	1p36.12
PLA2G6	Phospholipase A2, Group Vi	AR	PARK14	603604	22q13.1
PNKD	Pnkd Metallo-Beta-Lactamase Domain-Containing Protein	AD	DYT 8	609023	2q35
PNPLA6	Patatin-Like Phospholipase Domain-Containing Protein 6	AR	spastic paraplegia 39, Boucher-Neuhauser syndrome	603197	19p13.2
POLG	Polymerase, Dna, Gamma	AR	POLG-Related Disorders	174763	15q26.1
PRKCG	Protein Kinase C, Gamma	AD	SCA14	176980	19q13.42
PRKRA	Protein Kinase, Interferon-Inducible Double-Stranded Rna-Dependent Activator	AR	DYT 16	603424	2q31.2

PRNP	Prion Protein	AD	Huntington disease-like 1	176640	20p13
PRRT2	Proline-Rich Transmembrane Protein 2	AD	DYT 10, episodic kinesigenic dyskinesia 1	614386	16p11.2
PSEN1	Presenilin 1	AD	Alzheimer's disease	104311	14q24.2
RAB39B	Rab39b, Member Ras Oncogene Family	XLD	Waisman Syndrome	300774	Xq28
SACS	Sacsin	AR	Autosomal Recessive Spastic Ataxia of Charlevoix-Saguenay	604490	13q12.12
SCARB2	Scavenger Receptor Class B, Member 2	AR	Action Myoclonus Renal Failure Syndrome	602257	4q21.1
SCN2A	Sodium Voltage-Gated Channel, Alpha Subunit 2	AD	episodic ataxia 9	182390	2q24.3
SCN4A	Sodium Voltage-Gated Channel, Alpha Subunit 4;	AD	Myotonia congenita	603967	17q23.3
SETX	Senataxin	AR	Ataxia with Oculomotor Apraxia Type 2	608465	9q34.13
SGCE	Sarcoglycan, Epsilon	AD	DYT 11	604149	7q21.3
SIL1	Sil1 Nucleotide Exchange Factor	AR	Marinesco-Sjogren syndrome	608005	5q31.2
SLC1A3	Solute Carrier Family 1 (Glial High Affinity Glutamate Transporter), Member 3	AD	Episodic ataxia 6	600111	5p13.2
SLC20A2	Solute Carrier Family 20 (Phosphate Transporter), Member 2	AD	Idiopathic basal ganglia calcification 1	158378	8p11.21
SLC2A1	Solute Carrier Family 2 (Facilitated Glucose Transporter), Member 1	AD	DYT 9	138140	1p34.2
SLC39A14	Solute Carrier Family 39 (Zinc Transporter), Member 14	AR	Hypermanganesemia with dystonia 2	608736	8p21.3
SLC6A3	Solute Carrier Family 6 (Neurotransmitter Transporter, Dopamine), Member 3	AR	*SLC6A3*-Related Dopamine Transporter Deficiency Syndrome	126455	5p15.33
SNCA	Synuclein, Alpha	AD	PARK1/4	163890	4q22.1

SNX14	Sorting Nexin 14	AR	Spinocerebellar ataxia, autosomal recessive 20	616105	6q14.3
SPR	Sepiapterin Reductase	AR	Sepiapterin reductase deficiency	182125	2p13.2
SPTBN2	Spectrin, Beta, Nonerythrocytic, 2	AD	SCA5	604985	11q13.2
SQSTM1	Sequestosome 1	AD	FTD-ALS	601530	5q35.3
SYNJ1	Synaptojanin 1	AR	PARK20	604297	21q22.11
TARDBP	Amyotrophic Lateral Sclerosis 10 With Or Without Frontotemporal Dementia	AD	FTD-ALS	612069	1p36.22
TBK1	Tank-Binding Kinase 1	AD	FTD-ALS	604834	12q14.2
TENM4	Teneurin Transmembrane Protein 4	AD	Essential tremor 5	610084	11q14.1
TGM6	Transglutaminase 6	AD	SCA35	613900	20p13
TH	Tyrosine Hydroxylase	AR	DYT 5b	191290	11p15.5
THAP1	Thap Domain-Containing Protein 1	AD	DYT 6	609520	8p11.21
TMEM230	Transmembrane Protein 230	AD	PARK21	617019	20p13-p12.3
TMEM240	Transmembrane Protein 240	AD	SCA21	616101	1p36.33
TOR1A	Torsin 1a	AD	DYT 1	605204	9q34.11
TTBK2	Tau Tubulin Kinase 2	AD	SCA11	611695	15q15.2
TTPA	Tocopherol Transfer Protein, Alpha	AR	Ataxia with isolated vitamin E deficiency	600415	8q12.3
TUBB4A	Tubulin, Beta-4a	AD	DYT 4	602662	19p13.3
UBQLN2	Ubiquilin 2	AD	FTD-ALS	300264	Xp11.21
UCHL1	Ubiquitin Carboxyl-Terminal Esterase L1	AR	PARK5	191342	4p13
VCP	Valosin-Containing Protein	AD	FTD-ALS	601023	9p13.3
VPS13A	Vacuolar Protein Sorting 13 Homolog A	AR	Choreoacanthocytosis	605978	9q21.2
VPS13C	Vacuolar Protein Sorting 13 Homolog C	AR	PARK23	608879	15q22.2

VPS35	Vps35 Retromer Complex Component	AD	PARK17	601501	16q11.2
WDR45	Wd Repeat-Containing Protein 45	XLD	Neurodegeneration with brain iron accumulation 5	300526	Xp11.23
XK	Kell Blood Group Protein, Mcleod Syndrome-Associated	XLR	McLeod syndrome	314850	Xp21.1
XPR1	Xenotropic And Polytropic Retrovirus Receptor	AD	idiopathic basal ganglia calcification 6	605237	1q25.3

Abbreviations: AD: autosomal dominant inherited mode; AR: autosomal recessive inherited mode; CBS: corticobasal syndrome; FTD-ALS: frontotemporal dementia-amyotrophic lateral sclerosis; PSP: progressive supranuclear palsy; SCA: spinocerebellar ataxia; XLD: X-linked dominant inherited mode; XLR: X-linked recessive inherited mode

Supplementary Table 7. The regions with sequencing depth below 20x in the target sequencing and whole exome sequencing

Chromosome	Name	Start	End	%GC	Mean depth (x)
Sequencing of 116 targeted genes					
chr1	*TMEM240*	1475832	1475952	83.47%	0.394737
chr1	*ATP13A2*	17338145	17338325	80.11%	16.42105
chr1	*ATP13A2*	17338327	17338567	79.67%	18.86842
chr1	*HPCA*	33351985	33352165	79.01%	11.44737
chr11	*DRD2*	113345774	113346014	78.84%	5
chr14	*CCDC88C*	91884077	91884257	80.11%	19.53947
chr19	*DNMT1*	10341830	10342010	76.24%	8.947368
chr19	*CACNA1A*	13617144	13617264	78.51%	19.15789
chr3	*ANO10*	43663249	43663489	76.35%	11.61842
chr5	*SLC6A3*	1445413	1445593	73.48%	16.30263
chr8	*SLC39A14*	22224721	22224961	79.67%	15.89474
chr9	*SETX*	135230247	135230427	74.59%	10.31579
chrX	*CASK*	41782492	41782732	79.67%	3.815789
Whole exome sequencing					
chr3	*ADCY5*	123001142	123003583	52.50%	11.29
chr18	*AFG3L2*	12359925	12360050	43.65%	14.44
chr18	*AFG3L2*	12376967	12377275	79.29%	9.59
chr11	*ANO3*	26210669	26210877	37.80%	0.44
chr11	*ANO3*	26331191	26331266	44.74%	0
chr11	*ANO3*	26495448	26495675	39.04%	0.2
chr11	*ANO3*	26681808	26684836	35.19%	9.81
chr3	*ANO10*	43663264	43663418	78.06%	0.31
chr3	*ANO10*	43663400	43663569	78.24%	0.8
chr3	*ANO10*	43733008	43733086	64.56%	0.79
chr9	*APTX*	32985968	32986028	52.46%	3.17
chr9	*APTX*	32997240	32997346	47.66%	0
chr9	*APTX*	33024801	33025118	63.84%	0.09
chr9	*APTX*	33024997	33025118	64.75%	0
chr9	*APTX*	33025020	33025118	63.64%	0
chr11	*ATM*	108093558	108093913	63.48%	1.2
chr11	*ATM*	108094255	108094413	49.06%	0
chr11	*ATM*	108095510	108095598	38.20%	0
chr11	*ATM*	108236051	108239826	37.82%	6.07
chrX	*ATP6AP2*	40464812	40465888	32.22%	9.84
chr17	*CACNA1G*	48638075	48639062	75.61%	10.26
chr2	*CACNB4*	152689285	152695893	31.81%	7.29
chr2	*CACNB4*	152826794	152826940	38.78%	0

chr2	*CACNB4*	152828399	152828566	55.36%	2.29
chrX	*CASK*	41374188	41379849	42.02%	3.84
chr7	*CHCHD2*	56169254	56169554	37.21%	11.58
chr7	*CHCHD2*	56169265	56169554	37.24%	12.01
chr3	*CHMP2B*	87302861	87304698	30.74%	8.75
chr3	*CHMP2B*	87302861	87304706	30.72%	8.71
chr9	*CIZ1*	130953710	130953851	80.28%	0.49
chr9	*CIZ1*	130953710	130953868	79.87%	0.53
chr9	*CIZ1*	130966464	130966662	68.84%	0.46
chr17	*COASY*	40714091	40714237	57.82%	13.03
chr2	*COL6A3*	238322595	238322850	52.73%	1.69
chr2	*DCTN1*	74618920	74619214	68.47%	0.26
chr3	*DNAJC13*	132136345	132136604	71.54%	2.79
chr3	*DNAJC13*	132136503	132136604	70.59%	2.47
chr3	*DNAJC13*	132191400	132191415	31.25%	0.45
chr3	*DNAJC13*	132224145	132224259	30.43%	14.1
chr1	*DNAJC6*	65811293	65811406	44.74%	0
chr1	*DNAJC6*	65878606	65881552	35.32%	7.04
chr1	*DNAJC6*	65878606	65881554	35.30%	7.03
chr11	*DRD2*	113345797	113346001	79.02%	0
chr11	*DRD2*	113345797	113346120	82.41%	0
chr3	*DRD3*	113894772	113895040	37.92%	0.34
chr3	*DRD3*	113897503	113897623	45.45%	0.35
chr3	*DRD3*	113897503	113897899	45.09%	0.12
chr3	*DRD3*	113918119	113918254	49.26%	0
chr3	*EIF4G1*	184032282	184032462	74.59%	0
chr3	*EIF4G1*	184032289	184032462	74.14%	0
chr6	*ELOVL4*	80624531	80626600	32.90%	6.76
chr6	*ELOVL5*	53132195	53134068	35.81%	15.46
chr6	*ELOVL5*	53132201	53134068	35.71%	15.51
chr13	*FGF14*	102373204	102375317	39.50%	13.58
chr13	*FGF14*	102480071	102480272	29.21%	0
chr13	*FGF14*	102845386	102845713	46.65%	0.16
chr13	*FGF14*	102845472	102845713	48.76%	0.13
chr13	*FGF14*	102858216	102858327	54.46%	0.27
chr13	*FGF14*	102873046	102873283	45.38%	0.31
chr13	*FGF14*	102946323	102946390	42.65%	0
chr13	*FGF14*	102962820	102963036	42.40%	0.13
chr13	*FGF14*	102969985	102970054	58.57%	0.13
chr13	*FGF14*	103018794	103018963	52.35%	0
chr13	*FGF14*	103046107	103046181	52.00%	0.25
chr13	*FGF14*	103054303	103054382	47.50%	0.12

chr13	*FGF14*	103054472	103054778	50.81%	0.27
chr13	*FGF14*	103054476	103054778	50.83%	0.27
chr13	*FGF14*	103054485	103054778	51.02%	0.27
chr16	*FUS*	31202719	31206192	45.94%	2.5
chr9	*FXN*	71650478	71650863	67.62%	15.51
chr9	*FXN*	71687527	71693992	43.49%	5.67
chr9	*FXN*	71687527	71693993	43.50%	5.67
chr1	*GBA*	155214296	155214653	62.57%	0.22
chr2	*GIGYF2*	233562014	233562102	62.92%	1.41
chr2	*GIGYF2*	233565294	233565364	43.66%	0
chr2	*GIGYF2*	233568133	233568199	41.79%	0
chr2	*GIGYF2*	233589283	233589401	56.30%	0.65
chr2	*GIGYF2*	233595831	233595944	42.98%	0
chr2	*GIGYF2*	233599126	233599196	57.75%	0.25
chr2	*GIGYF2*	233600472	233601358	31.23%	0.33
chr2	*GIGYF2*	233721502	233725287	43.03%	10.62
chr18	*GNAL*	11751470	11751507	50.00%	0.09
chr18	*GNAL*	11751470	11751705	68.22%	0.53
chr18	*GNAL*	11857436	11857784	51.86%	0.51
chr4	*GRID2*	93225116	93225895	53.59%	17.1
chr17	*GRN*	42422453	42422702	56.80%	0.13
chr1	*HPCA*	33352091	33352116	73.08%	0.36
chr7	*IFRD1*	112063198	112063487	41.72%	0
chr7	*IFRD1*	112092112	112092566	45.49%	0.62
chr7	*IFRD1*	112115196	112115290	56.84%	1.47
chr7	*IFRD1*	112115484	112117258	36.17%	3.01
chr3	*ITPR1*	4535031	4535289	63.71%	3.22
chr3	*ITPR1*	4536114	4536190	59.74%	0
chr3	*ITPR1*	4887822	4889524	33.41%	6.37
chr12	*KCNA1*	5019070	5019638	65.73%	0.13
chr1	*KCND3*	112531369	112531777	58.19%	0.77
chr22	*KCTD17*	37458564	37459430	62.75%	9.62
chr12	*LRRK2*	40761445	40763086	29.29%	3.44
chr17	*MAPT*	43971747	43972052	76.14%	0.32
chr3	*MCCC1*	182746880	182746977	39.80%	0
chr3	*MCCC1*	182816275	182817031	50.46%	3.73
chrX	*MECP2*	153323849	153324017	55.62%	0
chr1	*MR1*	181002560	181002600	39.02%	0.75
chr1	*MR1*	181006132	181006248	45.30%	0
chr1	*MR1*	181024360	181031074	44.24%	5.08
chr20	*PANK2*	3869485	3869551	68.66%	1.27
chr20	*PANK2*	3871890	3872058	53.85%	1.32

chr20	*PANK2*	3903890	3910529	50.38%	4.09
chr1	*PARK7*	8021713	8021795	68.67%	0.8
chr1	*PARK7*	8021770	8021853	69.05%	0.95
chr22	*PDGFB*	39619363	39621318	54.50%	0.48
chr22	*PDGFB*	39639905	39641060	69.46%	9.53
chr20	*PDYN*	1973229	1973289	57.38%	0.71
chr20	*PDYN*	1973229	1973452	54.02%	1.44
chr20	*PDYN*	1974332	1974394	52.38%	0
chr20	*PDYN*	1974556	1974704	62.42%	0.04
chr20	*PDYN*	1974556	1974931	61.70%	0.32
chr10	*PHYH*	13341419	13341746	67.38%	1.13
chr22	*PLA2G6*	38577670	38577837	61.31%	0
chr22	*PLA2G6*	38577790	38577916	62.20%	0
chr2	*PNKD*	219209530	219211516	57.73%	8.23
chr19	*PNPLA6*	7599037	7599125	68.54%	0.4
chr19	*PNPLA6*	7599127	7599332	57.77%	0.03
chr19	*PNPLA6*	7599590	7599788	58.29%	0
chr19	*PNPLA6*	7599635	7599788	57.14%	0
chr19	*PNPLA6*	7600012	7600088	67.53%	3.5
chr15	*POLG*	89877903	89878026	76.61%	0.46
chr15	*POLG*	89877915	89878026	77.68%	0.45
chr19	*PRKCG*	54410247	54410901	60.76%	13.04
chr2	*PRKRA*	179315288	179315355	67.65%	8.67
chr20	*PRNP*	4666739	4667154	63.70%	0.52
chr20	*PRNP*	4666739	4667158	63.81%	0.52
chr20	*PRNP*	4667151	4667382	78.45%	0.82
chr16	*PRRT2*	29823408	29823644	74.26%	1.23
chr16	*PRRT2*	29823408	29823672	73.96%	1.18
chr16	*PRRT2*	29823513	29823644	70.45%	0.78
chr16	*PRRT2*	29825653	29827202	57.29%	13.32
chr16	*PRRT2*	29825948	29827202	55.94%	5.04
chr14	*PSEN1*	73603142	73603291	66.00%	0.35
chr14	*PSEN1*	73603214	73603291	66.67%	0.13
chr14	*PSEN1*	73685841	73690399	41.96%	3.47
chr13	*SACS*	24007753	24007841	62.92%	0.15
chr4	*SCARB2*	77079891	77082904	37.69%	6.75
chr2	*SCN2A*	166095923	166096150	35.96%	0
chr2	*SCN2A*	166150340	166150645	37.91%	0.06
chr2	*SCN2A*	166153526	166153645	36.67%	15.41
chr2	*SCN2A*	166237602	166237707	37.74%	10.22
chr9	*SETX*	135229035	135229142	45.37%	0.48
chr9	*SETX*	135230037	135230243	73.91%	0

chr9	*SETX*	135230303	135230373	70.42%	0
chr7	*SGCE*	94227241	94227316	61.84%	13.53
chr7	*SGCE*	94270208	94270276	62.32%	0.64
chr5	*SIL1*	138532129	138532217	47.19%	0
chr5	*SIL1*	138533957	138534057	72.28%	0.24
chr5	*SIL1*	138533957	138534065	72.48%	0.26
chr5	*SLC1A3*	36606456	36606837	49.21%	0
chr5	*SLC1A3*	36606707	36606837	52.67%	0
chr5	*SLC1A3*	36686166	36688436	39.81%	7.42
chr8	*SLC20A2*	42358548	42358749	42.08%	0.14
chr8	*SLC20A2*	42396594	42396655	70.97%	0
chr8	*SLC20A2*	42396963	42397356	69.04%	0
chr8	*SLC39A14*	22224742	22224921	77.78%	0.26
chr8	*SLC39A14*	22224790	22224921	78.03%	0.32
chr8	*SLC39A14*	22225036	22225250	73.95%	0.16
chr8	*SLC39A14*	22236939	22237027	58.43%	0.34
chr8	*SLC39A14*	22248242	22248360	56.30%	0.23
chr8	*SLC39A14*	22250342	22250745	54.95%	0.8
chr8	*SLC39A14*	22250342	22250932	55.84%	1.08
chr8	*SLC39A14*	22250566	22250745	59.44%	0.95
chr8	*SLC39A14*	22277064	22280249	42.97%	13.81
chr5	*SLC6A3*	1445462	1445555	76.60%	0.41
chr4	*SNCA*	90645249	90647811	33.63%	4.98
chr4	*SNCA*	90757893	90758127	55.32%	0
chr4	*SNCA*	90758112	90758350	68.62%	0.82
chr4	*SNCA*	90759402	90759447	50.00%	0
chr6	*SNX14*	86217685	86218031	37.75%	14.98
chr6	*SNX14*	86227725	86227766	30.95%	3.33
chr6	*SNX14*	86253322	86253478	29.30%	14.56
chr6	*SNX14*	86267693	86267778	33.72%	13.29
chr6	*SNX14*	86277251	86277295	37.78%	16.87
chr6	*SNX14*	86290913	86290976	43.75%	0
chr6	*SNX14*	86303551	86303874	66.98%	7.31
chr5	*SQSTM1*	179234002	179234123	78.69%	0.36
chr5	*SQSTM1*	179238573	179238682	49.09%	0.2
chr21	*SYNJ1*	34048641	34048665	44.00%	13.56
chr21	*SYNJ1*	34100147	34100250	77.88%	10.12
chr1	*TARDBP*	11072678	11072800	72.36%	4.28
chr12	*TBK1*	64845899	64845967	78.26%	0
chr12	*TBK1*	64890146	64890186	26.83%	8.76
chr11	*TENM4*	78859754	78859851	29.59%	0.02
chr11	*TENM4*	78926852	78926954	58.25%	0.46

chr11	*TENM4*	79008532	79008588	47.37%	0
chr11	*TENM4*	79151552	79151695	76.39%	0
chr8	*THAP1*	42691816	42693479	32.21%	15.31
chr20	*TMEM230*	5080483	5081577	38.00%	8.21
chr20	*TMEM230*	5080495	5081577	39.00%	8.3
chr20	*TMEM230*	5086833	5086956	48.00%	18.93
chr20	*TMEM230*	5092145	5092251	57.00%	0
chr20	*TMEM230*	5092409	5092454	39.00%	0.13
chr15	*TTBK2*	43030927	43038455	38.36%	15.34
chr15	*TTBK2*	43212635	43212976	74.85%	0.48
chr8	*TTPA*	63972047	63973984	33.64%	10.39
chr8	*TTPA*	63998376	63998612	76.37%	13.87
chr9	*VCP*	35071753	35072305	75.41%	2.85
chr9	*VPS13A*	79820893	79820995	32.04%	12.09
chr9	*VPS13A*	79997194	79997921	32.97%	3.7
chr9	*VPS13A*	79999500	80000106	26.52%	4.01
chr9	*VPS13A*	80030871	80032399	33.09%	7.49
chr9	*VPS13A*	80030871	80036453	35.00%	2.13
chr15	*VPS13C*	62144589	62146757	33.66%	12.31
chr15	*VPS13C*	62156504	62160146	29.04%	1.34
chr15	*VPS13C*	62315609	62315719	27.93%	2.3
chr16	*VPS35*	46691890	46694563	39.27%	10.82
chr16	*VPS35*	46710494	46710604	38.74%	11.79
chrX	*WDR45*	48937409	48937546	72.46%	0.26
chrX	*WDR45*	48957987	48958059	69.86%	0.67
chr1	*XPR1*	180805657	180808004	33.48%	13.84
chr1	*XPR1*	180853141	180859415	36.88%	4.18
