# Supplementary material for: The genetic spectrum of a cohort of patients clinically diagnosed as Parkinson’s disease in mainland China
Source: NPJ Parkinsons Dis. 2023 May 17;9:76. doi: 10.1038/s41531-023-00518-9 (PMC10192372; doi:10.1038/s41531-023-00518-9)
Supplement: Supplementary file 2 — Reporting Summary [file 41531_2023_518_MOESM2_ESM.pdf]

nature portfolio

Corresponding author(s): Yu An; Jian-Jun Wu; Jian Wang

Last updated by author(s): Mar 22, 2023

Reporting Summary

Nature Portfolio wishes to improve the reproducibility of the work that we publish. This form provides structure for consistency and transparency in reporting. For further information on Nature Portfolio policies, see our Editorial Policies and the Editorial Policy Checklist.

Statistics

For all statistical analyses, confirm that the following items are present in the figure legend, table legend, main text, or Methods section.

n/a	Confirmed	
☐	☒	The exact sample size (*n*) for each experimental group/condition, given as a discrete number and unit of measurement
☐	☒	A statement on whether measurements were taken from distinct samples or whether the same sample was measured repeatedly
☐	☒	The statistical test(s) used AND whether they are one- or two-sided<br>*Only common tests should be described solely by name; describe more complex techniques in the Methods section.*
☐	☒	A description of all covariates tested
☐	☒	A description of any assumptions or corrections, such as tests of normality and adjustment for multiple comparisons
☐	☒	A full description of the statistical parameters including central tendency (e.g. means) or other basic estimates (e.g. regression coefficient) AND variation (e.g. standard deviation) or associated estimates of uncertainty (e.g. confidence intervals)
☐	☒	For null hypothesis testing, the test statistic (e.g. *F*, *t*, *r*) with confidence intervals, effect sizes, degrees of freedom and *P* value noted<br>*Give P values as exact values whenever suitable.*
☒	☐	For Bayesian analysis, information on the choice of priors and Markov chain Monte Carlo settings
☒	☐	For hierarchical and complex designs, identification of the appropriate level for tests and full reporting of outcomes
☒	☐	Estimates of effect sizes (e.g. Cohen's *d*, Pearson's *r*), indicating how they were calculated

Our web collection on statistics for biologists contains articles on many of the points above.

Software and code

Policy information about availability of computer code

Data collection	No software was used.
Data analysis	The statistical analysis was conducted by STATA 17.0 (StataCorp). The bioinformatic analysis of the sequncing data was conducted according to the previously reported sequencing analysis pipline, which is available at https://www.protocols.io/view/adbs-whole-exome-sequencing-wes-analysis-pipeline-8epv56rz6g1b/v1.

For manuscripts utilizing custom algorithms or software that are central to the research but not yet described in published literature, software must be made available to editors and reviewers. We strongly encourage code deposition in a community repository (e.g. GitHub). See the Nature Portfolio guidelines for submitting code & software for further information.

Data

Policy information about availability of data

All manuscripts must include a data availability statement. This statement should provide the following information, where applicable:

- Accession codes, unique identifiers, or web links for publicly available datasets
- A description of any restrictions on data availability
- For clinical datasets or third party data, please ensure that the statement adheres to our policy

The data that support the findings of this study, including the raw DNA sequencing data generated during the current study, are available from the corresponding author Jian Wang upon request. Restrictions may be applied to sensitive data for privacy preservation.

Human research participants

Policy information about studies involving human research participants and Sex and Gender in Research.

Reporting on sex and gender	In Chinese culture, sex (biological attribute) and gender (shaped by social and cultural circumstances) were consistent, so the disaggregated sex and gender data wasn't collected. Sex of participants was determined based on biological concept. Participants in this study were enrolled regardless of sex. Three hundred and fifty-two female participants and 480 male participants were enrolled. Sex- and gender-based analyses weren't performed, and our findings applied to both males and females.
Population characteristics	See below.
Recruitment	Patients meeting the following criteria from February 2014 to December 2020 were investigated retrospectively in the movement disorder clinic of Huashan Hospital: (1) a diagnosis of PD at the initial clinical visit; (2) AAO <50, or AAO >=50 but with family history of PD (defined as have at least one other affected relative in the family); and (3) consent to genetic testing related to PD. The diagnostic criteria used for PD were the UK PD Society Brain Bank Clinical Diagnostic Criteria (for patients recruited before 2016) or the 2015 MDS Clinical Diagnostic Criteria for PD (for patients recruited from 2016 on)
Ethics oversight	The study was approved by the Institutional Review Board of Huashan Hospital and the China human genetic resources management office. Written informed consent was obtained from all study participants.

Note that full information on the approval of the study protocol must also be provided in the manuscript.

Field-specific reporting

Please select the one below that is the best fit for your research. If you are not sure, read the appropriate sections before making your selection.

☐ Life sciences ☒ Behavioural & social sciences ☐ Ecological, evolutionary & environmental sciences

For a reference copy of the document with all sections, see nature.com/documents/nr-reporting-summary-flat.pdf

Behavioural & social sciences study design

All studies must disclose on these points even when the disclosure is negative.

Study description	This study was a quantitative study.
Research sample	A total of 832 participants were continually enrolled from the movement disorder clinic of Huashan Hospital, Fudan University. Patients meeting the following criteria were investigated retrospectively: (1) a diagnosis of PD at the initial clinical visit; (2) AAO <50, or AAO >=50 but with family history of PD (defined as have at least one other affected relative in the family); and (3) consent to genetic testing related to PD. The diagnostic criteria used for PD were the UK PD Society Brain Bank Clinical Diagnostic Criteria (for patients recruited before 2016) or the 2015 MDS Clinical Diagnostic Criteria for PD (for patients recruited from 2016 on). Among them, 42.31% (352/832) were female. The median age of the participants at examination was 48.00 (16.50) years. The sample is representative. Early-onset and familial PD patients were chosen as genetic factors may play an important role in these patient's onset.
Sampling strategy	Convenience sampling was used in this study as the sampling method. No sample size calculation was performed. The sample size was sufficient based on similar genetic studies conducted before.
Data collection	The clinical assessments were performed through a face-to-face interview with all patients. Baseline data were collected including demographic profiles (age, gender, education), disease history, family history, clinical signs, comorbidities, medications, and neurological examination results. An established method was used to calculate the levodopa equivalent daily dosage (LEDD). Data of the participants were collected by pen and paper, for self-reported and evaluation of two senior investigators of movement disorders. The results were than updated to the database for storage. The researcher was blind during data collection.
Timing	The participants were enrolled from February 2014 to December 2020.
Data exclusions	Nine participants were excluded during the statistical analysis of UPDRS III scores (off), because they could not tolerate the withdrawal of anti-Parkinson's disease medications
Non-participation	No participants declined participation.
Randomization	Participants were not allocated into experimental groups.

Reporting for specific materials, systems and methods

We require information from authors about some types of materials, experimental systems and methods used in many studies. Here, indicate whether each material, system or method listed is relevant to your study. If you are not sure if a list item applies to your research, read the appropriate section before selecting a response.

Materials & experimental systems

n/a	Involved in the study
☒	☐ Antibodies
☒	☐ Eukaryotic cell lines
☒	☐ Palaeontology and archaeology
☒	☐ Animals and other organisms
☒	☐ Clinical data
☒	☐ Dual use research of concern

Methods

n/a	Involved in the study
☒	☐ ChIP-seq
☒	☐ Flow cytometry
☒	☐ MRI-based neuroimaging
